# Global transcriptional response after exposure of fission yeast cells to ultraviolet light

**DOI:** 10.1186/1471-2121-10-87

**Published:** 2009-12-16

**Authors:** Henriette C Skjølberg, Øyvind Fensgård, Hilde Nilsen, Beáta Grallert, Erik Boye

**Affiliations:** 1 Department of Cell Biology, Institute for Cancer Research, Oslo University Hospital, Radiumhospitalet, Montebello, 0310 Oslo, Norway; 2 Institute for Molecular Biosciences, University of Oslo, Blindernveien 31, 0371 Oslo, Norway; 3 The Biotechnology Center, University of Oslo, Gaustadalleen 21, 0349 Oslo, Norway

## Abstract

**Background:**

In many cell types, including the fission yeast *Schizosaccharomyces pombe*, a set of checkpoints are induced by perturbations of the cell cycle or by DNA damage. Many of the checkpoint responses include a substantial change of the transcriptional pattern. As part of characterising a novel G1/S checkpoint in fission yeast we have investigated whether a transcriptional response is induced after irradiation with ultraviolet light.

**Results:**

Microarray analyses were used to measure the global transcription levels of all open reading frames of fission yeast after 254 nm ultraviolet irradiation, which is known to induce a G1/S checkpoint. We discovered a surprisingly weak transcriptional response, which is quite unlike the marked changes detected after some other types of treatment and in several other checkpoints. Interestingly, the alterations in gene expression after ultraviolet irradiation were not similar to those observed after ionising radiation or oxidative stress. Pathway analysis suggests that there is little systematic transcriptional response to the irradiation by ultraviolet light, but a marked, coordinated transcriptional response was noted on progression of the cells from G1 to S phase.

**Conclusion:**

There is little response in fission yeast to ultraviolet light at the transcriptional level. Amongst the genes induced or repressed after ultraviolet irradiation we found none that are likely to be involved in the G1/S checkpoint mechanism, suggesting that the checkpoint is not dependent upon transcriptional regulation.

## Background

Cell cycle progression is fundamental for all proliferation. Transition from one cell-cycle phase to the next is often brought about by a changed transcriptional pattern: repression of specific genes and/or expression of new genes promote progression from one phase into the next. The regulation of transcriptional patterns during the cell cycle is conserved from yeast to humans [1,2], although the actual genes and transcriptional factors involved are not necessarily conserved. In addition to transcriptional regulation the key components of the cell cycle are also frequently regulated at the translational and the post-translational levels.

Regulation of transcription is important in the cellular response to environmental stress. Exposure to radiation, toxic chemicals, fluctuations in temperature, osmolarity or nutrient availability profoundly affect cell growth and the genomic expression programme is adjusted to adapt to the different challenges. Microarray technology has been used to characterise global gene expression profiles for several different stress conditions in the model organism *Schizosaccharomyces pombe *[3,4]. A common set of genes responding to many different forms of stress has been identified in both fission and budding yeast. These genes are known as core environmental stress response genes, CESR [3] in *S. pombe *and environmental stress response genes [5] or the common environmental response genes [6] in *Saccharomyces cerevisiae*. In addition to this common pattern there are genes that are specifically expressed in response to each individual stress treatment.

In general, stress represents a threat to genome stability. Depending on the type of damage inflicted and the position in the cell cycle different strategies are used for handling a stress situation. Checkpoint mechanisms delay the cell cycle to allow the cells to repair DNA damage and to ensure stable inheritance of the genome. Several checkpoint pathways target the transcription machinery to ensure the appropriate expression levels of genes involved in the response to the insult. In *S. pombe *there are separate checkpoints that inhibit mitosis when the DNA is damaged (the G2/M checkpoint) or when S phase has not been completed (the S/M checkpoint) and that inhibits DNA replication when the DNA is damaged (the intra-S checkpoint) [7]. These checkpoints have at least two features in common: they all operate through the five so-called checkpoint Rad proteins and they all bring about the cell-cycle delay via inhibition of the Cdc2 protein kinase, the key regulator of cell-cycle progression [8]. In addition, they also include Rad3-dependent transcriptional responses [4,9,10].

In G1 phase the cell decides whether to commit to a new round of the cell cycle or to enter stationary phase or meiosis. The G1/S DNA damage checkpoint regulates the transition into S phase [11], and insensitivity to growth-inhibitory signals, especially in the G1 phase, is one of the hallmarks of cancer [12]. We have recently discovered and partly characterised a novel checkpoint mechanism in *S. pombe *which delays S-phase entry after UVC irradiation in a Gcn2-dependent manner [13]. In the present work we have investigated whether the response to UVC in G1 phase involves a specific transcriptional response and searched for possible genes to be involved in the G1/S checkpoint. Furthermore, we compare the genes differentially expressed after UVC irradiation with the transcriptional response to oxidative stress (H_2_O_2_) and ionising radiation (IR).

## Results

We have performed genome-wide expression analyses of UVC-irradiated G1-phase fission yeast cells to further characterise the G1/S checkpoint [13,14]. We have investigated what kind of transcriptional response UVC irradiation imposes on the cells and searched for potential candidate genes involved in the regulatory process of the G1/S checkpoint. The cells were synchronised by employing a temperature-sensitive version of Cdc10, a transcription factor required for progression from G1 into S phase. This method gives good synchrony and allows convenient detection of the G1/S checkpoint. During a four-hour shift to 36°C the cells were arrested in G1 phase and could be released synchronously into the cell cycle or kept in G1 phase. Total RNA was isolated from both UVC-irradiated cells and unirradiated control cells. The RNA was subjected to total genomic microarray analysis.

### The two experiments

Two distinct experiments were performed (see also Additional file 1): First, G1-phase cells were irradiated and kept at the restrictive temperature (hereafter called the "restrictive-temperature experiment"). Thirty minutes after the time of irradiation the UVC-irradiated (UV30) and unirradiated control (C30) cells were collected. These cells were still in early G1 phase due to the continued inactivation of Cdc10. Second, G1-cells were released into the cell cycle after synchronisation by reducing the temperature to 25°C, thus reactivating Cdc10, allowing the cells to continue in the cell cycle. Cell samples were collected at 0, 30 and 90 min after irradiation (hereafter called the "time-course experiment"). Samples of irradiated and unirradiated cells were analyzed on microarrays and compared to a common reference pool (see Methods). Flow cytometry (Additional file 2) demonstrated that the control cells had entered S phase by 60 minutes after release, whereas the UVC-irradiated cells delayed in G1 phase and moved from G1 to S phase around 90 minutes after release, in agreement with previous data [13].

We have previously shown the existence of the G1/S checkpoint using several synchronisation methods [14], but none of the other methods provided good enough synchrony to perform similar analyses on global transcription. We have looked at our data for all known Cdc10 targets in the 12 arrays from the time-course experiment and observed no trend showing that UVC delays the occurrence of these transcripts. This is consistent with our RNA blots of the two selected transcripts *cdc18 *and *cig2*, which are not delayed by UVC in block-and-release experiments [Fig. 4D in [14]].

### The restrictive-temperature experiment

**Figure 1:**
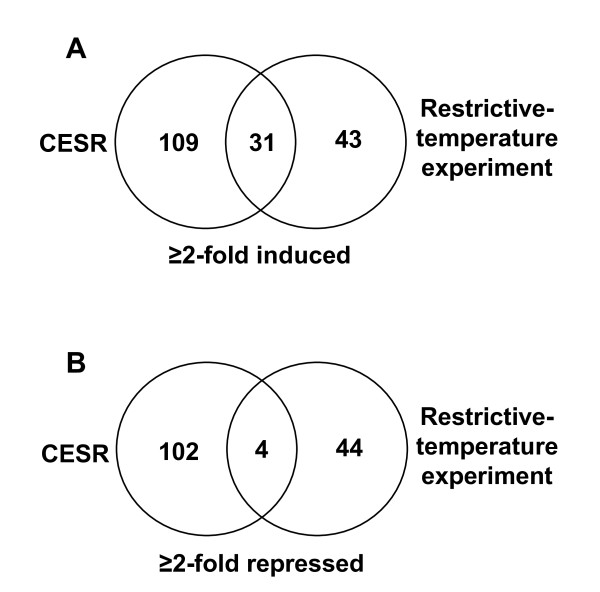
**Comparison of induced and repressed genes from the restrictive-temperature experiment and CESR genes**. The number of genes induced (A) and repressed (B) more than two-fold in the restrictive-temperature experiment illustrated in a Venn diagram. The numbers of genes common for the restrictive-temperature experiment and the previously identified CESR are shown within the overlapping regions.

In this experiment we searched for genes that altered their expression more than twofold as a consequence of UVC irradiation in G1 phase. Of almost 5000 genes represented on the microarrays as few as 74 genes were induced twofold or more and 43 of these were non-CESR genes (Fig. 1A). Most of the 74 genes were induced two- to threefold, and only three genes were induced more than fivefold. Most of the induced non-CESR genes are likely to be UVC-specific and not cell-cycle related, since the cells did not move into S phase during the time of the experiment. No non-CESR genes were found to be induced by both UVC and H_2_O_2 _[3], but two genes, *SPCC132.04c *and *SPBC16A3.17c *were induced after both UVC (this work) and IR treatment [4] (see Table 1). We categorised the 43 non-CESR genes into eight different groups according to the functions of their products (Table 1). These genes are involved in a variety of functions such as signalling and stress response, ribosome biogenesis and translation, DNA/RNA binding, and as many as 15 of the genes are involved in transport mechanisms. There were no obvious candidates for genes involved in the G1/S checkpoint amongst the 74 upregulated genes. Surprisingly, only one of the induced genes, *rhp4b*, encoding a nucleotide excision repair factor, is involved in DNA repair of UVC-induced lesions, strongly suggesting that the capacity to perform DNA repair is not regulated at the transcriptional level. Another possibly interesting induced gene is *pyp1*, a protein-tyrosine phosphatase that acts on Sty1, the MAPK (mitogen-activated protein kinase) that regulates various stress responses in *S. pombe *[15]. We have shown that the G1/S checkpoint does not require Sty1 [13], but it is possible that Pyp1 has additional targets in the cell besides Sty1.

**Table 1:** UV-induced genes not present in the CESR

UV-induced genes not present in the CESR at UV30:	
**Gene name**	**Annotation**

*gst2*	Glutathione S-transferase, similarity to Gst1p, induced by oxidative stress. Also induced by IR
*SPBC1A4.07c*	Sof1-like domain containing family and contains 7 WD domains, similarity to *S. cerevisiae *Sof1p
*rrn3*	Involved in initiation of transcription of rDNA promoter
*scw1*	Involved in negative regulation of cell wall integrity and septum formation
*SPBC19F5.02c*	Protein containing six WD domains, similarity to S. cerevisiae Utp4p
*SPCC584.07c*	Pseudogene
*SPAC17A2.02c*	Protein of unknown function, similarity to uncharacterized C. albicans Ipf15301p
*SPBC8E4.02c*	Protein of unknown function
fip1	Iron permease FTR1 family. Also indused after IR and between C30 and C90

Gene annotations are from GeneDB http://www.genedb.org/genedb/pombe/index.jsp.

We also identified 44 genes that were repressed more than twofold and almost all of them (40) were non-CESR genes (Fig. 1B). These 40 genes were categorised according to the function of their products (Additional file 3), and, like for the proteins encoded by the induced genes, were known to be involved in a wide variety of activities including transport, metabolism, mating and mitochondrial activities. Amongst the repressed genes no obvious candidate genes for checkpoint regulators were found.

### The time-course experiment

In the unirradiated control cells only 53 genes from the whole dataset were upregulated (more than twofold) and 29 were downregulated in either the C30 and/or the C90 sample relative to the situation in cells at the start of the experiment (C0) (Table 2). In comparison, altogether 41 genes were upregulated and 35 downregulated in either the UV0, UV30 or the UV90 sample relative to C0 during the time-course. Like in the restrictive-temperature experiment, most of the induced genes were only upregulated two- or threefold. Only four genes in either C or UV were induced more than fivefold in both repeats of the experiment and this was only found for the C90 sample. It is striking that only about 1% of around 5000 genes has a changed expression after cell cycle progression and UVC irradiation.

**Table 2:** Differentially expressed genes in the time-course experiment

The time course experiment:		
**Differentially expressed genes**		

	**induced ≥2-fold**	**repressed ≥2-fold**
UV0	0	0
UV30	12	3
UV90	35	34

Total	41	35

C0		
C30	13	8
C90	41	28

Total	53	29

**Differentially expressed genes when comparing the different timepoints**		

**Comparison**	**induced (P ≤ 0.05)**	**induced ≥2-fold**
UV0-UV30	11	0
UV30-UV90	0	0
UV0-UV90	241	12

Total	241	12

CO-C30	4	0
C30-C90	47	14
CO-C90	129	30

Total	143	20

Furthermore, we searched for genes differentially expressed between 0/30, 30/90 and 0/90 minutes in both the C and UV samples. To this end we used moderated t-statistics with a P-value cut-off of 0.05 (for details see Methods). In these experiments each gene could potentially be assigned 12 expression values (three time points, C and UV, two repeats). About 35% of the values were missing in the entire dataset. The reasons for the missing values will be discussed below. We decided to remove the data for a gene if four or more of the 12 possible data values were missing. This action reduced the dataset from 5266 to 2836 genes, and data for the resulting 54% of the genes was considered more reliable and was used for further analysis.

### Cell-cycle-regulated genes

**Figure 2:**
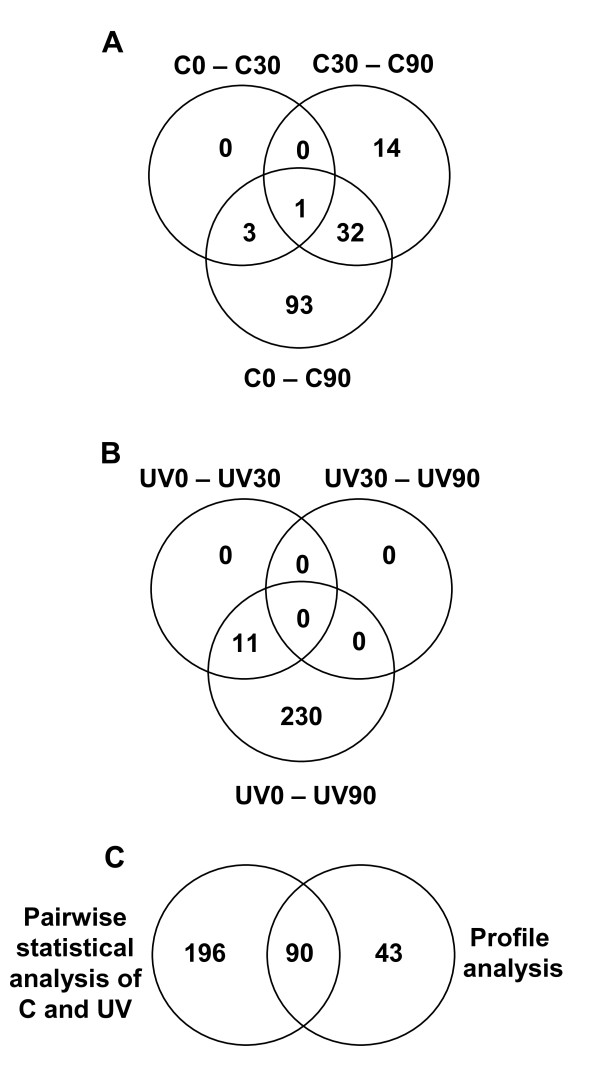
**Differentially expressed genes during the time-course experiment**. Venn diagram comparison of differentially expressed genes in control cells when comparing C0 to C30, C30 to C90 and C0 to C90 (A), and in UVC-irradiated cells when comparing UV0 to UV30, UV30 to UV90 and UV0 to UV90 (B). The numbers of differentially expressed genes in either the control [C] or irradiated [UVC] cells identified by the pairwise statistical and the profile analysis are illustrated in a Venn diagram (C). The numbers of common genes are shown within the overlapping regions.

The RNA samples from irradiated cells reflect gene expression changes that occur for two separate reasons: first, the cells are progressing through the cell cycle and will necessarily change gene expression [16] and, second, the cells have been exposed to UVC light and will display stress-related and UVC-specific changes. In contrast, differences between the samples in the restrictive-temperature experiment (above) should reflect only the stress-related and UVC-specific changes. In the present analysis, we attempt to identify the genes specifically affected by UVC exposure. For this reason we identified the genes in unirradiated control cells whose expression varied after release into the cell cycle and these genes were classified as not specifically responding to UVC. In the unirradiated cells altogether 143 genes were found to be differentially expressed between G1 phase (C0 or C30) and S/G2 phase (C90) (Fig. 2A and Table 2). Only 4 genes (3+1) were differentially expressed at 30 minutes after release into the cell cycle (C0 compared to C30). In comparing C30 (late G1 phase) with C90 (S/G2 phase) 47 genes (32+14+1) were found to be differentially expressed. Finally, 129 genes (93+32+1) had a changed transcriptional level when comparing C0 (early G1 phase) to C90 (S/G2 phase). This means that, not surprisingly, most of the induced genes were found in cells entering S phase (the comparison of C0 with C90 and C30 with C90). Well-known G1-specific genes, like *cdc18*, *cig2*, *cdc22 *and *cdt2*, were induced normally during G1 phase (C30) in our study (data not shown), serving as convenient controls.

### UVC-regulated genes

Using the P-value cut-off of 0.05, we identified as many as 241 genes in total that were differentially expressed in the UVC-irradiated cells after release into the cell cycle (Fig. 2B and Table 2). Of all the 241 genes only 11 were different between UV0 and UV30, so little was happening at the transcriptional level during the first 30 minutes after irradiation. No genes were determined to be differentially expressed when comparing UV30 (late G1 phase) and UV90 (S phase). However, as many as 230 genes were defined to be different between UV0 (early G1 phase) and UV90 (S phase). The apparent discrepancy between the numbers of regulated genes between UV0/UV30 and UV30/UV90 on the one hand and between UV0/UV90 on the other can be explained as follows: Expression of a number of genes is different between UV0 and UV30, but only 11 genes were significantly different (P < 0.05). Similarly, a number of genes changed their expression between UV30 and UV90, but no difference passed the threshold we had set. However, when comparing UV0 and UV90, a number of genes (230) had altered their expression sufficiently during the total interval. It follows that the level of change for all of these genes was low.

Some of the 241 genes that were up- or downregulated after UVC in this experiment are regulated as a consequence of the cell-cycle progression and not of the UVC irradiation. To identify the UVC-specific transcripts, we excluded the 143 cell-cycle-regulated genes identified above (see *Cell-cycle-regulated genes*), resulting in 172 UVC-regulated genes (Additional file 4), of which 162 are non-CESR genes. Of these 162 genes as many as 26 genes are dedicated to the translational machinery. Nine of the 162 genes were specifically upregulated in UV30 (Table 3), which is at a time when cells are arrested in G1 phase by the G1/S checkpoint. These 9 genes were compared to the set of non-CESR genes induced by H_2_O_2 _and IR [3,4]. No genes were found to be induced by both UVC and H_2_O_2 _treatment. Only two genes, *gst2 *and *fip1*, were induced after both UVC (this work) and IR treatment. None of the 9 UVC-specific genes are likely to be regulators of the G1/S checkpoint, judging from their annotations.

**Table 3:** UV-induced genes not present in the CESR at UV30

UV-induced genes not present in the CESR:	
**Gene name**	**Annotation**

Repair and DNA metabolism	
*rhp4b*	Nucleotide excision repair factor involved in the repair of UV damaged DNA
*mus81*	Holliday junction resolvase subunit that associates with Eme1p
Metabolism	
*SPCC132.04c*	Similarity to S. cerevisiae Gdh2p, a glutamate dehydrogenase
Transort	
*SPAC328.09*	Similarity to 2-oxodicarboxylate transporter (S. cerevisiae Odc2p
*SPCC794.04c*	Sugar (and other) transporter family and the major facilitator superfamily
*str1*	Probable ferrichrome-iron transporter
*SPCPB1C11.01*	Similarity to S. cerevisiae Mep2p, ammonium transporter family of membrane transporters
*SPAC1002.16c*	Sugar (and other) transporter family, and the major facilitator superfamily
*SPBC36.02c*	Similarity to C. albicans Flu1p, a membrane transporter, major facilitator superfamily
*SPBC530.15c*	Similarity to S. cerevisiae Tpo3p, a polyamine transport protein
*SPBC409.08*	Similarity to S. cerevisiae Tpo2p, a polyamine transport protein
*SPAC8C9.12c*	Similarity to mitochondrial RNA splicing protein 3 (S. cerevisiae Mrs3p), mitochondrial carrier protein family
*ptr2-a;ptr2*	Similarity to S. cerevisiae Ptr2p, a peptide permease nitrogen-repressible transporter
*SPCC569.05c*	Major facilitator superfamily, similarity to C. albicans Flu1p
*SPBC16A3.17c*	Major facilitator superfamily, similarity to S. pombe Fnx1p, transporter required for long-term survival in N starved cells
*SPCC2H8.02*	Major facilitator superfamily and the sugar (and other) transporter family
*SPCC2H8.00*	Major facilitator superfamily and the sugar (and other) transporter family, similarity to S. cerevisiae Pho84p
*SPCC1183.11*	Mechanosensitive ion channel family
Signaling and stress response	
*SPAC9B6.03*	Protein containing a FYVE zinc finger domain, which bind phosphatidylinositol 3-phosphate
*gaf2*	Iron-sensing transcription factor that binds GATA elements to regulate iron transporter gene transcription
*rst2*	Transcriptional activator that positively regulates the transcription of ste11 and fbp2
*SPBC19C2.13c*	Similarity to S. cerevisiae Ncs2p, involved in pseudohyphal growth and cellular response to starvation
*pyp1*	Protein-tyrosine phosphatase that acts on Sty1p and negatively regulates mitosis
*(isa1)*	Protein that binds ferredoxin (Etp1p) and contains iron-sulfur clusters
*(cta3)*	Probable Ca2+-ATPase, transcription is induced under high salt conditions
Ribosome biogenesis and translation	
*rrn2*	Protein involved in initiation of transcription of rDNA promoter
*SPCP1E11.06*	Similarity to S. cerevisiae Nsa2p, a nuclear protein involved in ribosome biogenesis, part of the small ribosomal subunit
DNA/RNA binding	
*SPBP8B7.15c*	Zinc knuckle domain, which can bind RNA or DNA in eukaryotes, similarity to S. cerevisiae Mpe1p
*cbh2*	DNA binding protein, may be involved in chromosome segregation
Others	
*SPAC323.07c*	Member of the MatE family, which are integral membrane proteins
*ppr1*	Resistance to the L-proline analog AZC, catalyzes acetylation of AZC, homolog of S. cerevisiae Mpr1p
*SPAC3H8.09c*	Containing an RNA recognition motif, similarity to S. cerevisiae Nab3p
*SPBC1773.17c*	Containing D-isomer specific 2-hydroxyacid dehydrogenase NAD binding and catalytic domains
*SPAPB18E9.04c*	Similarity to S. cerevisiae Pry3p, may have a role in mating efficiency
Protein of unknown function	
*SPAPB18E9.03c*	*SPNCRNA.101 SPAC18G6.09c SPAPB1A10.06 SPBC19C7.04c SPAC17A5.8* *SPAC23H3.15c* *SPCC2H8.01 SPAC8C9.10c*

Gene annotations are from GeneDB http://www.genedb.org/genedb/pombe/index.jsp.

### Profile analyses

To further analyse the kinetics of gene expression in the time-course experiment we applied the software package maSigPro [17] on the filtered data, which allowed a multiple comparison of all three time points. There were 133 genes that significantly changed their expression levels during the time-course (P ≤ 0.05), and the expression of almost all (105 genes) was changed in both control and UVC-irradiated cells. Fifteen genes were identified as specific for the irradiated cells. There was a good correlation between the genes identified in this profile analysis and the differentially expressed genes determined in the statistical analysis above (Fig. 2C).

**Figure 3:**
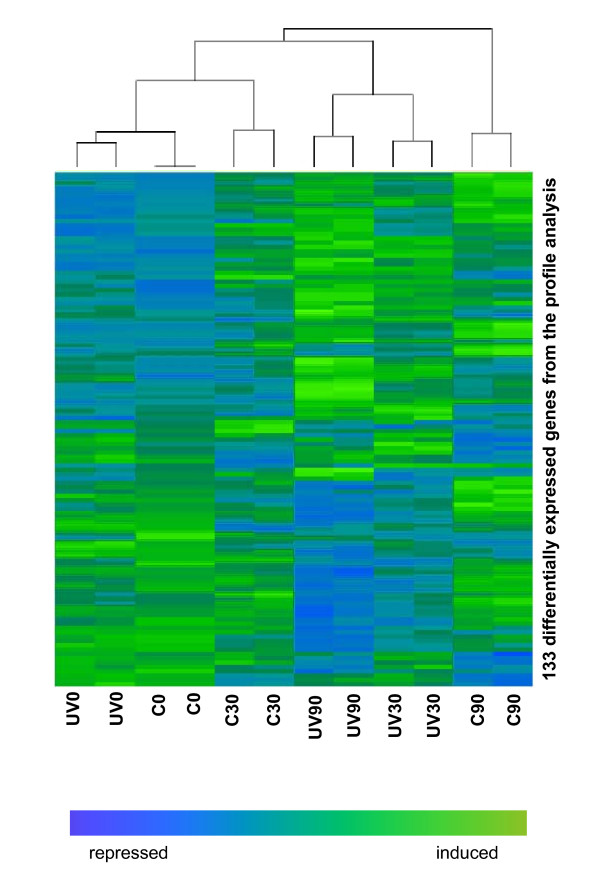
**Regulation of gene expression during the time-course experiment in control and UVC-irradiated cells**. The expression pattern of 133 genes whose expression changed significantly (P ≤ 0.05) during the time course are shown. The columns represent samples taken after 0, 30 or 90 min in control and UVC-irradiated cells. Hierarchical clustering was performed as described in Methods, pairing the 133 genes in the different samples according to their expression level. The changes in transcription level are colour coded with induced genes as green and repressed genes as blue.

The expression levels of the 133 genes with expression that varied during the time-course were subjected to a clustering analysis, forming a two-dimensional map (Fig. 3). Genes being up- or downregulated at the particular time point are represented by different colours, green indicating induction and blue repression. The map shows that analysis of the two biological replicates revealed that they were indeed similar and assembled into the closest branches of the cluster. This confirms a good reproducibility of the experiments. Furthermore, the profile analysis is also consistent with the conclusions of the pairwise statistical analyses (above), showing that the expression patterns in UV30 and UV90 were similar. The map shows little difference between control and irradiated samples at all time points, and the least closely related branches of the cluster refer to changes occurring during progression in the cell cycle. Thus, this analysis corroborates our conclusions from the above pairwise comparisons, that the cell cycle progression affects transcription profiles more than the UVC treatment and there is only a weak transcriptional response to UVC irradiation.

### Pathway analyses

**Figure 4:**
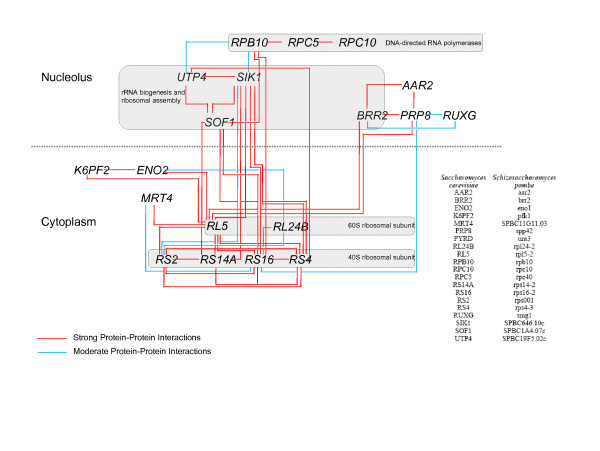
**Protein-protein interaction network**. Gene products of the regulated genes from the time-course experiment form an interconnected network involving translation and transcription. Protein-protein interactions were analyzed in FunCoup using the corresponding *S. cerevisiae *orthologues (presented in the table on the right). Strong (red lines) and moderate (blue lines) interactions are shown. DNA-directed RNA polymerases, 60S and 40S ribosomal subunits and genes involved in rRNA biogenesis and ribosomal assembly are indicated by grey boxes. The full list of interactions is found in Additional file 7.

Gene ontology (GO) enrichment analysis was performed on the 43 UVC-induced genes in the restrictive-temperature experiment (Table 1) using the DAVID software after ID-conversion. Ten unique genes (20%) were members of enriched GO groups, suggesting upregulation of genes involved in ion transport or ion homeostasis in the restrictive-temperature experiment (Additional file 5). Analysis of over-represented GO annotations amongst the 172 upregulated genes in the time-course experiment (Table 2) showed that 38 unique genes (22%) are members of the enriched GO groups. The nature of these groups indicates regulation of genes that affect protein biosynthesis or structural components of ribosomes (Table 4). Further network analysis shows a concerted response involving direct protein-protein interactions between 20 of the 172 induced gene products. Thus, the analyses suggest a coordinated induction of rRNA biogenesis, ribosome assembly and components of the 60S and 40S ribosomal subunits in the time-course experiment (Fig. 4). (Additional file 7)

**Table 4:** Enriched Gene Ontology Groups in the restrictive-temperature experiment

Category	GO number	Term	Count*	%	P-value
GOTERM_Cellular Component	GO:0005887	integral to plasma membrane	3	8.11	0,024
	GO:0044459	plasma membrane part	4	10.81	0,035
	GO:0031226	intrinsic to plasma membrane	3	8.11	0,038
					
GOTERM_Biological Process	GO:0015674	di-, tri-valent inorganic cation transport	4	10.81	0,001
	GO:0006812	cation transport	5	13.51	0,007
	GO:0030001	metal ion transport	4	10.81	0,008
	GO:0030003	cellular cation homeostasis	4	10.81	0,018
	GO:0055082	cellular chemical homeostasis	4	10.81	0,020
	GO:0050801	ion homeostasis	4	10.81	0,022
					
GOTERM_Molecular Function	GO:0008324	cation transmembrane transporter activity	5	13.51	0,011
	GO:0046873	metal ion transmembrane transporter activity	3	8.11	0,042

* Count denotes the number of genes in our dataset in each cluster. Percent coverage of these genes relative to the numbers on the gene ontology clusters were calculated.

### Confirmation of the microarray data

**Figure 5:**
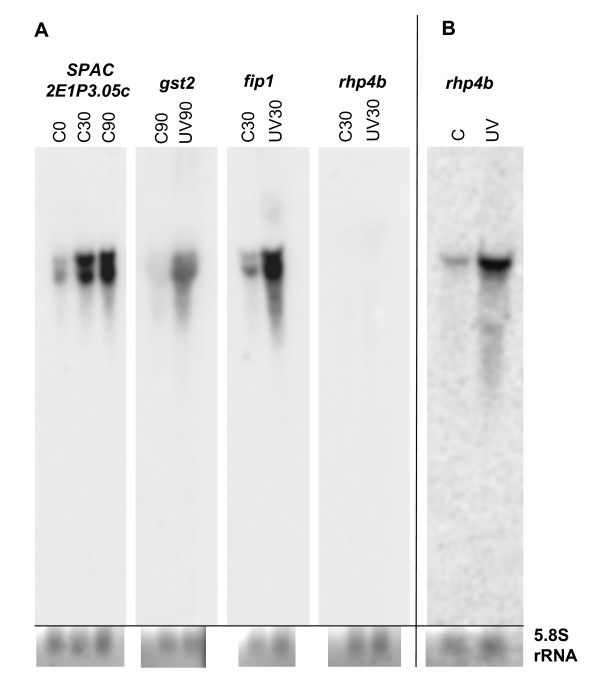
**Detection of selected transcripts by RNA blotting and hybridisation**. Four different transcripts from different time points and treatments in the time-course experiment (A) and one transcript from the restrictive-temperature experiment (B) were detected as described in Methods. The 5.8S rRNA from the ethidium bromide stained agarose gel before blotting was used as a loading control.

RNA blotting and hybridisation was used to confirm our microarray results for four selected transcripts. The induction of *SPAC2E1P3.05c*, *fip1 *and *gst2 *found in the time-course experiment was verified (Fig. 5A). Fip1, an iron permease, and Gst2, a glutathione S-transferase, have also been shown to be induced after IR [4]. On the other hand, *rhp4b *had no values in any of the 12 arrays in our time-course experiments and was also not detected after RNA blotting and hybridisation. However, in the restrictive-temperature experiment, *rhp4b *transcription was found to be induced by UVC irradiation and this finding was also confirmed by RNA blotting (Fig. 5B). Therefore, RNA-blotting experiments with all four selected genes verified the results from the microarray experiments.

### UVC does not induce the unfolded protein response

The unfolded protein response (UPR) is activated by the accumulation of unfolded proteins in the endoplasmic reticulum (ER). The UPR triggers a transcriptional response which serves to induce the production of ER components and to increase the degradation capacity to dispose of the unfolded proteins [18]. It is possible that UVC irradiation might stress the ER and thus activate the UPR. Furthermore, GCN2 has been shown to be required for the induction of a majority of UPR target genes during ER stress in *S. cerevisiae *[19]. We therefore used the present data from both the restrictive-temperature and time-course experiments to investigate whether UPR genes are induced by UVC in fission yeast. We identified UPR-genes in fission yeast as the homologues of the UPR-induced budding yeast genes [20]. There was little, if any, induction of the UPR-genes identified by this method (Additional file 6). The lack of transcriptional response of these genes after UVC strongly argues that the G1/S checkpoint is not a manifestation of the UPR.

## Discussion

Here we have investigated gene expression in *S. pombe *cells traversing the G1/S border in a synchronous manner, both UVC-irradiated and unirradiated cells. The transcriptional response after UVC-irradiation in G1 phase was surprisingly weak. The vast majority of genes did not change their transcription pattern appreciably and the few that did increased or decreased their expression levels only two- to three-fold.

### Comparison of the data from the two experiments

162 genes were identified as specifically UVC-regulated in the time-course experiment and 43 in the restrictive-temperature experiment. Surprisingly, as few as 4 genes (see Additional file 4) were found to be common for the two datasets, and these 4 genes were not differently expressed in UV0 and UV30 in the time-course experiment (Table 3), which is the time period when the irradiated cells were arrested in the G1/S checkpoint. The UVC irradiation elicits a quite weak transcriptional response on the cells both when considering the number of genes affected and the level of the response for the affected genes. Thus, our assay must be considered to be rather sensitive and even a small change in the experimental setup might bring the marginal levels of gene expression over or under our threshold, which could be one reason for the poor overlap between the two experiments. This further underlines our conclusion that UVC has only a marginal effect on gene transcription when given in G1 phase. It is possible that the biological differences between the cells in our two types of experiments is dominating and that the dissimilar sets of regulated genes reflect a biological difference rather than an artefact of our data analysis. In the restrictive-temperature experiment cells were arrested in G1 phase by the inactivation of Cdc10, which means that cell-cycle dependent responses were inhibited. Thus, this experiment reveals transcriptional regulation exclusively due to UVC irradiation, at a specific stage in the cell cycle. In contrast, in the time-course experiment cells were allowed to progress into the cell cycle. Even though we have subtracted the cell-cycle-regulated genes when identifying the UVC-regulated genes, the cell cycle stage in which the cellular response is analysed was still different from that of cells held in the *cdc10 *block. Consistent with this line of reasoning, we have shown that little happened at the transcriptional level during the first 30 minutes after irradiation in the time-course experiment (only 11 regulated genes when comparing UV0 to UV30), and most of the 172 genes changed their transcription enough to satisfy the criteria of our analysis only by a later time point, as the cells entered S phase. Therefore these genes have not been identified as regulated in the restrictive-temperature experiment.

### Missing values

In the data for the time-course experiment values were missing for many data points. This can be attributed to at least two reasons: lack of expression of the relevant open reading frames and technical problems with the microarrays. At any one time there are genes that are poorly expressed, so it was expected that there would be some missing values in our datasets. Low expression is the most likely reason for the absent microarray signal for the *rhp4b *transcript, which could also not be detected after RNA blotting. However, lack of expression cannot explain all the missing values. The 12 microarrays used in the time-course experiment comprised from 18% to 55% missing values (on average 35%), arguing that there were technical problems with at least some of the microarrays. Such a high level and difference in the number of missing values was not found for the two repeats of the restrictive-temperature experiment, which both had about 6% missing values, further suggesting technical problems with the arrays used in the time-course experiment. It should be noted that the microarrays used for this experiment came from a different batch/production than those used for the restrictive-temperature experiment. This problem did reduce the quality of the data for some of the genes in the time-course experiment, and we decided to remove all the data pertaining to genes where too many data points were missing (detailed in Results). However, the stringency of our analysis allows us to draw conclusions in spite of the missing data. Furthermore, any technical problem would have affected a random set of genes, and a strong transcriptional response would have been obvious even from the time-course experiment.

### Comparisons with other organisms

This is the first report about the global transcriptional response after UVC irradiation in fission *S. pombe *and there are only a few reports about similar experiments in other organisms. The available data indicate that the weak transcriptional response to UVC stress we observed in fission yeast might be a conserved feature. For example, in human cells exposed to UVC only 155 of more than 7500 genes investigated changed their expression more than 2-fold [21]. An early microarray-report using *Escherichia coli *cells identified several differentially expressed genes after UVC-irradiation, but the response was generally not more than two-fold [22].

Many checkpoints, both in fission yeast and in other organisms, involve transcriptional regulation. For instance, in multicellular organisms one of the best characterised checkpoint targets is p53, a transcription factor which is mutated in over half of human cancers. p53 stimulates transcription of cell-cycle inhibitors such as p21 [23] and is essential for a persistent G1 arrest. Another tumour suppressor, pRb, targets the E2F-driven transcriptional programme in the G1/S checkpoint [23,24]. In budding yeast activation of the G1/S checkpoint also impinges on transcriptional regulation in that the transcription factor SWI6 is phosphorylated and thereby inactivated by the checkpoint protein RAD53, leading to delayed transcription of CLN1 and CLN2 and a delayed entry into S phase [25]. Recent data show that in fission yeast the transcriptional response is important in the intra-S checkpoint after exposure to hydroxyurea [10,26].

### Comparisons with other stress agents

We have observed no obvious change of the transcriptional programme that could be responsible for the G1/S checkpoint and also no strong induction of genes involved in DNA repair or checkpoint function. It is interesting to note that other DNA-damaging agents, such as H_2_O_2 _and IR, also do not lead totranscriptional induction of many DNA repair- or checkpoint-related genes [3,4]. Therefore, it is likely that there is no need to specifically induce transcription of DNA repair-related genes, suggesting that the DNA repair capacity is high already before the UVC exposure, as the case is for budding yeast [27].

We have compared our data from synchronised cells to data based on H_2_O_2_- and IR-treated asynchronous cells [3,4], and there is little overlap in the spectrum of non-CESR genes differentially expressed after exposure to the three agents. As discussed above, it seems that the cell-cycle position is important for the transcriptional profile obtained when exposing the cells to a stress treatment. However, when comparing the differentially expressed genes in asynchronously growing cells exposed to UVC (our unpublished observations) to cells exposed to H_2_O_2 _[3] and IR [4] there is little overlap.

### Pathway analyses

DNA-damaging agents, heat and other forms of stress give overlapping responses, described as the CESR, that involves 14% of the genome in *S. cerevisiae *[5]. Few UVC-specific expression changes have been reported [21] and the fold-changes observed are low [28]. It is therefore possible that transcriptional responses to heat stress combined with stringent statistical analyses and exclusion of the CESR-genes mask UVC-relevant gene expression changes in the restrictive-temperature experiment. Similarly, the absence of significant gene-expression changes after 30 minutes recovery in the time-course experiment indicates that UVC-relevant transcriptional responses are masked by the small fold-changes and extensive overlap between the UVC-specific response and the CESR. This is supported by appearance of differences on the transcriptional level first after 90 minutes. Our network analyses of gene-expression changes indicate that pathways involved in recovery after DNA damage are induced after 90 minutes (Fig. 4). The processes involved probably reflect the importance of the translation machinery in recovery after the insult. These recovery processes are likely to be important for survival after UVC treatment, as was shown in MMS-treated *S. cerevisiae *[29], although they may not be components of the G1/S checkpoint *per se*.

### Regulation of the G1/S checkpoint

In the G1/S checkpoint the Gcn2 kinase is activated to phosphorylate the eukaryotic initiation factor 2α, eIF2α, thereby inhibiting translation [13]. There is a good correlation between eIF2α phosphorylation and checkpoint activation [30], but it is still unclear whether and, if so, how the checkpoint is dependent upon this phosphorylation and on the ensuing downregulation of translation. Gcn2 is best known for its role in the starvation response, where eIF2α phosphorylation leads to induction of the transcription factor GCN4 both in budding yeast and higher eukaryotes. Fission yeast does not have a GCN4 homologue, and it remains to be seen whether the GCN2-dependent G1/S checkpoint in budding yeast [31] involves activation of GCN4. One might expect a transcriptional response after Gcn2 activation also in *S. pombe*, but the finding that no such transcriptional response could be identified argues that in fission yeast the G1/S checkpoint does not operate like it does in budding yeast or higher eukaryotes and is not dependent upon the strong transcriptional induction of one or a set of genes. We have shown that the G1/S checkpoint in *S. pombe *is associated with a strong downregulation of translation and it might be relevant that among the few genes that are affected at the transcription level, 15% affect the translation machinery. Importantly, amongst the transcriptionally regulated genes none were detected that are likely to be directly involved in the G1/S checkpoint according to their annotations.

Induction or inhibition of transcription is a fairly slow response and regulation of the cell cycle in *S. pombe *should preferably occur rapidly in order to be efficient and meaningful. Therefore, it intuitively makes sense that the present data suggest that the G1/S checkpoint is regulated at the level of protein modification and/or translation, which is rapid, rather than a slower regulation of gene expression.

## Conclusions

The transcriptional response to UVC irradiation of fission yeast cells in G1 phase was shown to be weak. We conclude that the novel G1/S checkpoint is not regulated by changing the transcriptional programme. This is supported by an examination of the few genes that are induced or repressed by UVC, and none of them appears to have any relationship to cell-cycle regulation.

## Methods

### Yeast strains and cell growth

The *cdc10-M17 *strain is a derivative of the L972 strain [32]. The basic growth media were as described [33]. The temperature-sensitive *cdc10 *cells were grown exponentially in EMM to an optical density (595 nm) of 0.15 (about 3 × 10^6 ^cells/ml), before they were synchronised by a four-hour temperature shift to 36°C and irradiated with 1100 J/m^2 ^UVC (254 nm), giving a cell survival of ~15% [14]. Samples of 25 ml of control or UVC-irradiated cells were harvested at different time points by centrifugation and snap-frozen in liquid nitrogen.

### RNA isolation

Total RNA was extracted using a hot phenol method [34]. RNA concentrations and qualities were measured in a NanoDrop (NanoDrop Technologies) and in a 2100 Bioanalyzer (Agilent Technologies).

### Microarray hybridization and data acquisition

Total RNA was reverse transcribed (GibcoBRL) in the presence of Cy3- or Cy5-labelled dCTP. The cDNA was hybridised onto glass DNA microarrays containing duplicate probes for 99.3% of all known and predicted open reading frames in the fission yeast genome (for details on protocols and microarrays, see Lyne et al. 2003 and http://www.sanger.ac.uk/PostGenomics/S_pombe). A GenePix 4000 B laser scanner was used for scanning the microarrays before analysis with GenePix Pro software (Axon Instruments). A Perl script was used for removing unreliable signals and normalization of the data [34]. All raw data are available under accession number A-MEXP-1666 and A-MEXP-1667 from ArrayExpress.

### Experimental design

Two different types of experiment were performed (see Results) and for both types the RNA from two biological repeats were analysed with a dye swap. Samples from all three time points of the "time-course experiment" were hybridised individually against a reference pool containing equal amounts of RNA from the unirradiated cells, at all the three time points (0, 30 and 90 min). After normalisation (for details see Lyne et al. 2003), the ratio of the values for the actual sample and for the reference pool for each gene was divided by the corresponding ratio for untreated cells at time 0 (0 min control/reference pool). Samples from UVC-irradiated cells kept at 36°C, the "restrictive temperature experiment", were hybridized to the arrays against RNA from unirradiated cells.

### Pathway analyses

Genes found differentially expressed in the time-course experiment (Table 4) were analyzed for gene ontology-enriched clusters using DAVID (Database for Annotation, Visualization and Integrated Discovery) http://niaid.abcc.ncifcrf.gov[35,36]. As *Schizosaccharomyces pombe *gene names are not recognized, the gene names were converted to UniProt accession numbers using the YOGY (eukarYotic OrtholoGY) software http://www.bahlerlab.info/YOGY/[37]. The YOGY software was also used to find *Saccharomyces cerevisiae *orthologues for use in the pathway analyses. When several orthologues were found, the *S. pombe *sequence was used as template for a BLAST search and the best hit in *S. cerevisiae *used. As there are very few resources available to investigate functional interactions in *S. pombe*, the *S. cerevisiae *orthologues were used to map protein-protein interactions. These interactions were processed using FunCoup (networks of functional coupling) http://funcoup.sbc.su.se/[38].

### Data evaluation

In both types of experiment genes were classified as differentially expressed when expression values were changed more than twofold (linear values) in both of the two biological repeats. Before statistically analysing the time-course experiment, a filtering of the data was performed. This filtering excluded all genes in the dataset that did not have a value in two-thirds of the arrays (i.e. missing more than 3 out of 12 values). The normalised expression values (see paragraph above) for the remaining genes were transformed from linear values to log2 values. Moderated t-statistics with a P-value cut-off of 0.05 was used to identify genes differently expressed during the time-course [39]. Benjamini and Hochberg's method was used to calculate adjusted P-values and to statistically correct for the occurrence of false positives [40]. The statistical analysis was performed using the programme R and Bioconductor [41]. The Bioconductor package maSigPro was used to perform profile analysis of the time-course experiment to show how gene expression changed with time. Benjamini and Hochberg's method was also used in the profile analysis and the P-value cut-off was set to 0.05.

### RNA blots

Total RNA was run on agarose gels in formaldehyde, blotted onto Hybond-XL membranes (Amersham Bioscience) and cross-linked by UVC. Probes were prepared by PCR of genomic DNA and labelled with ^32^P-d-CTP (Rediprime II Random prime labelling system, Amersham Bioscience). Phosphoimager screens were exposed to the washed blots and analyzed by a Pharos FX scanner (BioRad).

## Authors' contributions

HCS carried out all the experiments and drafted the manuscript. ØF and HN performed the pathway and GO analyses. BG and EB contributed in planning the project, in designing the experiments and in writing the manuscript. All authors have read and approved the final version of this manuscript.

## Supplementary Material

Additional file 1 **A schematic presentation of the experimental design**. Exponentially growing cells were synchronised by a four-hour temperature shift to 36°C. For the time-course experiment cells were UVC-irradiated when shifted back to the permissive temperature and control or irradiated cells were harvested at the time points indicated (black dots). For the restrictive-temperature experiment cells were UVC-irradiated at 36°C after synchronisation, held at the restrictive temperature and control or irradiated cells were harvested at the time point indicated (black dot). Click here for file

Additional file 2 **Flow cytometry histogram from the time-course experiment**. Flow cytometry histograms of control (C) and UVC-irradiated (UVC) G1-synchronised cells incubated for the times indicated (in minutes) after exposure. Click here for file

Additional file 3 **UV-repressed genes that are not CESR genes**. 44 genes that were repressed more than twofold were indentified and almost all of them (40) were non-CESR genes. These 40 genes were categorised according to the function of their products. Click here for file

Additional file 4 **172 genes changed in the time-course experiment by UV-irradiation**. The cell-cycle-regulated genes were excluded from the 241 genes that were up- or downregulated after UVC, in order to identify the UVC-specific transcripts, resulting in the 172 UVC-regulated genes shown here. Click here for file

Additional file 5 **Enriched gene ontology groups in the restrictive-temperature experiment**. Gene ontology (GO) enrichment analysis was performed on the 43 UVC-induced genes in the restrictive-temperature experiment (Table 1) using the DAVID software after ID-conversion. Ten unique genes (20%) were members of enriched GO groups. Click here for file

Additional file 7 **Genes induced in the time-course experiment: protein-protein interactions**. Gene products of the regulated genes from the timecourse experiment form an interconnected network involving translation and transcription. Protein-protein interactions were analyzed in FunCoup using the corresponding *S. cerevisiae *orthologues. Click here for file

Additional file 6 **Fission yeast homologues of UPR-induced budding yeast genes**. We used the data from both the restrictive-temperature and timecourse experiments to investigate whether UPR genes are induced by UVC in fission yeast. We identified UPR-genes in fission yeast as the homologues of the UPR-induced budding yeast genes. Click here for file
