# Supplementary figures and images for: Global transcriptional response after exposure of fission yeast cells to ultraviolet light
Source: BMC Cell Biol. 2009 Dec 16;10:87. doi: 10.1186/1471-2121-10-87 (PMC2806298; doi:10.1186/1471-2121-10-87)

Time-course experiment:

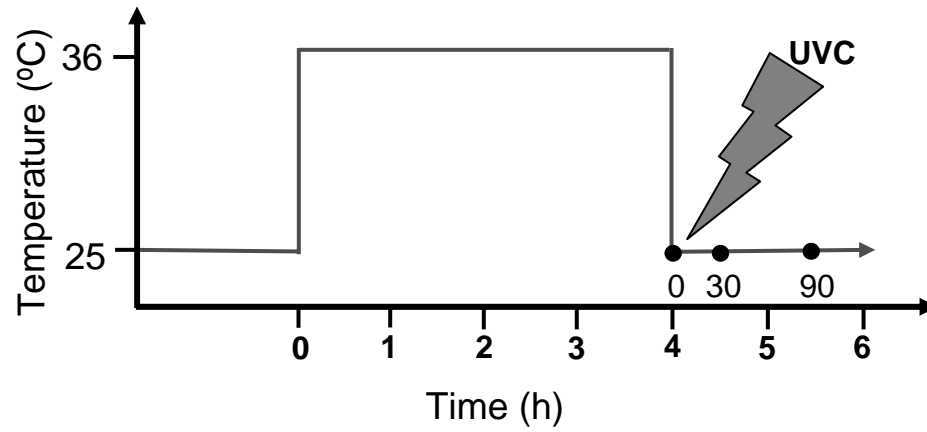

Restrictive-temperature experiment:

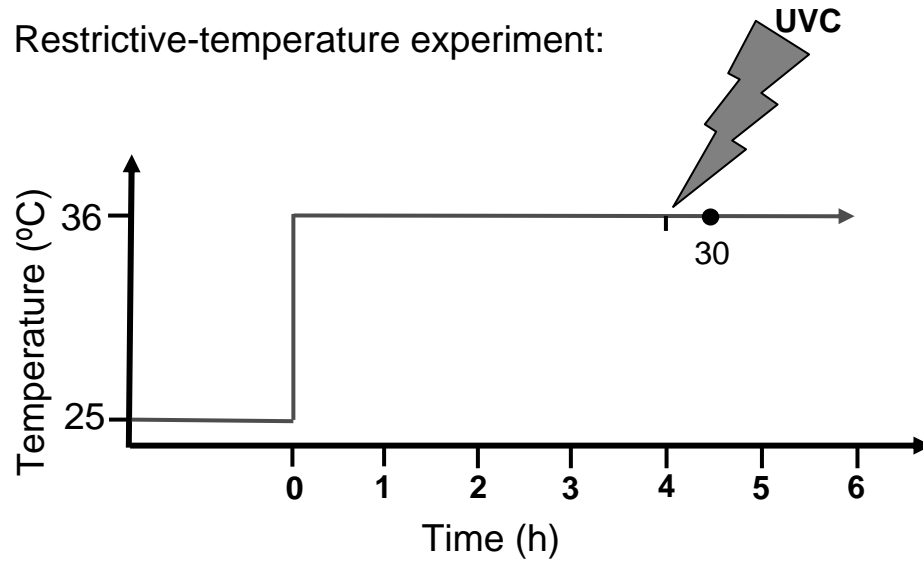

Supplement: Additional file 1 — A schematic presentation of the experimental design. Exponentially growing cells were synchronised by a four-hour temperature shift to 36°C. For the time-course experiment cells were UVC-irradiated when shifted back to the permissive temperature and control or irradiated cells were harvested at the time points indicated (black dots). For the restrictive-temperature experiment cells were UVC-irradiated at 36°C after synchronisation, held at the restrictive temperature and control or irradiated cells were harvested at the time point indicated (black dot). [file 1471-2121-10-87-S1.PDF]

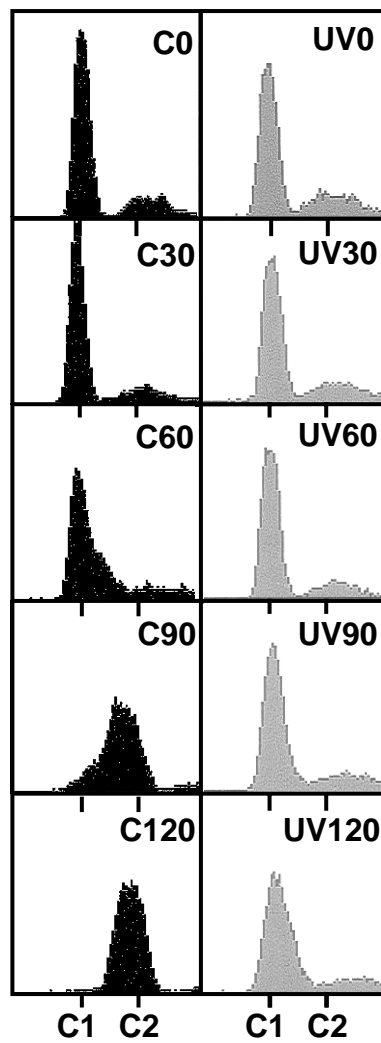

Supplement: Additional file 2 — Flow cytometry histogram from the time-course experiment. Flow cytometry histograms of control (C) and UVC-irradiated (UVC) G1-synchronised cells incubated for the times indicated (in minutes) after exposure. [file 1471-2121-10-87-S2.PDF]
