# Supplementary material for: Global transcriptional response after exposure of fission yeast cells to ultraviolet light
Source: BMC Cell Biol. 2009 Dec 16;10:87. doi: 10.1186/1471-2121-10-87 (PMC2806298; doi:10.1186/1471-2121-10-87)
Supplement: Additional file 3 — UV-repressed genes that are not CESR genes. 44 genes that were repressed more than twofold were indentified and almost all of them (40) were non-CESR genes. These 40 genes were categorised according to the function of their products. [file 1471-2121-10-87-S3.PDF]

UV-repressed genes that are not CESR genes			
**Transport**			
SPAC869.05c	Member of the sulfate transporter family		
SPBP4G3.01	Major facilitator superfamily, similarity to S. cerevisiae Pho84p		
SPBC1683.03c	Member of the major facilitator superfamily, similarity to uncharacterized S. cerevisiae Yor378p		
car1	Cell surface transporter involved in amiloride sensitivity		
SPBC1604.04	Member of the mitochondrial carrier protein family of membrane transporters		
SPBPB10D8.01	Prdicted cyctein transporter		
**Metabolism**			
SPAC869.02c	Similarity to C. albicans Yhb1p, required for metabolism and detoxification of nitric oxide		
SPBPB2B2.09c	Member of the ketopantoate reductase PanE or ApbA family, involved in thiamine biosynthesis		
SPAC3G9.11c	Similarity to pyruvate decarboxylase isozyme 3 (S. cerevisiae Pdc6p), which is a pyruvate decarboxylase		
SPAC4H3.08	Protein containing a short chain dehydrogenase domain		
SPAC9E9.09c	Protein with similarity to Emericella nidulans aldehyde dehydrogenase		
SPBC8E4.06c	Protein containing a copper amine oxidase domain		
**Mating**			
mfm1	Precursor polypeptide for the mating pheromone M factor that is produced by h- cells		
mfm3	Precursor polypeptide for the mating pheromone M factor that is produced by h- cells		
matmc: c1711.02	Mating type M-specific polypeptide Mc, transcription factor required for mating and meiosis		
rgs1	Regulator of G protein signaling (RGS) family member that negatively regulates the mating pheromone response pathway		
**Signaling and stress respons**			
SPBPB21E7.09	Similarity to L-asparaginase II (S. cerevisiae Asp3-2p), involved in the response to nitrogen starvation		
pho1	Non-specific acid phosphatase precursor		
**DNA/RNA binding**			
SPAC1039.05c	Protein containing two zinc finger C2H2 type repeats, which bind nucleic acids		
**Mitochondrial genes**			
cox1I1	Intron-encoded RNA maturase and DNA endonuclease encoded by the first intron of the mitochondrial cox1 gene		
urfA	Mitochondrially-encoded protein required for respiration, may be involved in maintenance or stability of the mitochondrial genome		
SPMIT.03	Protein possibly involved in mitochondrial splicing; encoded by an intron in the mitochondrial cox1 gene		
cobI	Protein with reverse transcriptase and reverse splicing activities, possibly involved in mitochondrial splicing		
cob	Cytochrome b, member of the cytochrome bc1 complex, mitochondrially encoded		
**non-coding RNA**			
misc_RNA_2.2.48	tos2	prl3	prl10
**Others**			
pmp20	Protein containing an AhpC or TSA family domain, similarity to peroxiredoxin 6		
pfk1	6-Phosphofructokinase beta subunit		
thi2	Thiazole biosynthetic enzyme		
SPBPB2B2.05	Protein containing a glutamine amidotransferase class-I domain and a peptidase C26 domain		
SPBPB2B2.06c	Protein containing a calcineurin-like phosphoesterase domain		
SPBPB8B6.03	Member of the amidase family, which catalyze hydrolysis of amides		
SPCC162.02c	Putative AMP-binding dehydrogenase		
SPBC1683.04	Similar to C. pseudotropicalis Bglp, a beta-glucosidase that catalyzes hydrolysis of cellobiose,		
SPBC1348.13	Pseudogen		
**Protein of unknown function**			
SPBPB7E8.01	SPAC27D7.11c	SPAC29B12.08	SPAC56F8.14c SPBC1347.11 SPAC1039.02 SPCC1235.12c
