# Supplementary material for: Global transcriptional response after exposure of fission yeast cells to ultraviolet light
Source: BMC Cell Biol. 2009 Dec 16;10:87. doi: 10.1186/1471-2121-10-87 (PMC2806298; doi:10.1186/1471-2121-10-87)
Supplement: Additional file 4 — 172 genes changed in the time-course experiment by UV-irradiation. The cell-cycle-regulated genes were excluded from the 241 genes that were up- or downregulated after UVC, in order to identify the UVC-specific transcripts, resulting in the 172 UVC-regulated genes shown here. [file 1471-2121-10-87-S4.PDF]

172 genes changed in the time-course experiment by UV-irradiation

Gene annotations are from GeneDB http://www.genedb.org/genedb/pombe/index.jsp.

Common induced genes in this table with Table1 (induced genes in the restrictive temperature experiment) are underlined.
The second pannel indicates if the gene is also induced in the CESR or after H2O2 or IR treatment.

**Gene name**		**Annotation**
ade3;min11		Putative phosphoribosylformylglycinamidine synthase
adh1		Alcohol dehydrogenase
ams2		Protein that binds binds chromatin at centromere GATA sequence and is involved in chromosome segregation
apc11		Component of the APC/cyclosome complex
arg5		Protein with high similarity to amidotransferase small subunit of carbamoylphosphate synthetase (*S. cerevisiae* Cpa1p), contains a glutamine amidotransferase class-I domain and a carbamoyl-phosphate synthase small chain CPSase domain
aur1		Aureobasidin A resistance protein, required for cell tip elongation
bgl2		Protein with high similarity to beta-glucosidase (*S. cerevisiae* Bgl2p), which is a cell wall endo-beta-1,3-glucanase, contains a glycosyl hydrolase family 17 domain
brr2		Component of the ribosome assembly chaperone (RAC) complex, which is required for pre-rRNA maturation, and a component of the Cdc5p-associated complex, which may be involved in pre-mRNA splicing
C1348.13		Pseudogene
ccr1		NADP-cytochrome P450 reductase
cnx1;cal1		Calnexin homolog involved in quality control process for secretory pathway proteins
coq3		Protein with moderate similarity to *S. cerevisiae* Coq3p, which is a 3-demethylubiquinone-9 3-methyltransferase that catalyzes the fourth and rate limiting step in coenzyme Q biosynthesis
coq7		Protein with high similarity to *S. cerevisiae* Cat5p, which is required for derepression of gluconeogenic enzymes and for coenzyme Q biosynthesis, member of the ubiquinone biosynthesis protein COQ7 family, which may be involved in energy generation
cox1		Cytochrome c oxidase subunit I, mitochondrially encoded
cox15		Electron transfer protein, contains a ferredoxin domain, has similarity to *S. cerevisiae* Cox15p and human COX15
cox1I1		Intron-encoded RNA maturase and DNA endonuclease encoded by the first intron of the mitochondrial cox1 gene
cox1I2b		Protein possibly involved in mitochondrial splicing; encoded by an intron in the mitochondrial cox1
cps1;bgs1		Putative component of 1,3-beta-D-glucan synthase, required for cell wall rigidity and septation
csk1		Cyclin-dependent kinase activating kinase (CAK) involved in activating Cdc2p cyclin-dependent kinase; activity is partially redundant with that of the Mcs6p-Mcs2p complex
CTOKYO_453.18		Conserved hypothetical gene
cut15		Putative importin alpha subunit
cyp51		Protein with high similarity to lanosterol 14alpha-demethylase (*P. carinii* ERG11), which is involved in resistance to some azole drugs, contains a cytochrome P450 domain
dis2: bws1		Serine-threonine protein phosphatase PP1-1; catalytic subunit of major type 1 protein
elf1		Protein involved in nuclear export of mRNA, member of the ATP-binding cassette (ABC) transporter family of ATPases
eno1		Enolase
exg3	*CESR*	*Protein with low similarity to exo-beta-(1,3)-glucanase (C. albicans Xog1p), which is involved in cell wall metabolism*
fkbp39		Putative peptidyl prolyl cis-trans isomerase, has moderate similarity to *S. cerevisiae* Npi46p
gpx1	*CESR*	*Glutathione peroxidase, may play a role in adaptation to oxidative stress*
gst2		Glutathione S-transferase with high similarity to Gst1p, induced by oxidative stress and involved in the cellular response to oxidative stress
his3		Histidinol-phosphate aminotransferase
its8		Protein involved in GPI anchor synthesis, cell wall integrity, and cytokinesis
leu3		Protein with high similarity to 2-Isopropylmalate synthase (*S. cerevisiae* Leu4p), which catalyzes the first step in the leucine biosynthesis pathway, member of the hydroxymethylglutaryl-CoA lyase (HMGL)-like family
lid2		Component of the Lid2C complex, which may interact with the Set1C complex, contains three PHD fingers, a helix-turn-helix BRIGHT domain, and a JmjC domain
lps1		tRNA pseudouridine synthase, converts uridine nucleosides to pseudouridine nucleosides in tRNA
lys3	*H2O2*	Probable saccharopine dehydrogenase
mal2		Protein required for centromere structure and function

mlo3		Protein containing one RNA recognition motif (RRM, RBD, or RNP), has low similarity to *S. cerevisiae* Yra1p, which has RNA:RNA annealing activity and is involved in mRNA packaging for export from the nucleus
ned1		Protein required for normal nuclear morphology and chromosome stability
obr1	*H2O2*	Ubiquinated histone-like protein that functions as an Rhp6p mediator in mating-type silencing, regulated by the Pap1p transcription factor
och1		Alpha-1,6-mannosyltransferase involved in the initiation of outer chain elongation of N-linked oligosaccharides
pcr1	*CESR*	*Transcription factor that plays roles in mating, meiosis and stress response, member of the bZIP-containing CREB/ATF family*
pfk1		6-Phosphofructokinase beta subunit
pmd1		ABC transporter involved in multidrug resistance
ppt1		Para-hydroxybenzoate--polyprenyltransferase, mitochondrial precursor
prl28		non-coding RNA
prl62		non-coding RNA
rad24		14-3-3 protein that inhibits meiosis and is involved in DNA damage checkpoint control and negative regulation of the Ras1p-Byr2p signaling pathway
rpb10		Shared subunit of RNA polymerase I, II, and III
rpc10		Shared subunit of RNA polymerases I, II, and III
rpc40		Putative shared subunit of RNA polymerases I and III
rpl18-1		60S ribosomal protein L18
rpl18-2		60S ribosomal protein L18
rpl24-2		60S ribosomal protein L24B
rpl30-2		Protein with strong similarity to S. pombe Rpl3001p, which is 60S ribosomal protein L30, contains a ribosomal protein L7Ae, L30e, S12e, or Gadd45 family domain
rpl5-2		60S ribosomal protein L5
rpl7-2		60S ribosomal protein L7B/L7-C
rps001		40S ribosomal protein S0
rps14-2		40S ribosomal protein S14
rps16-2		40S ribosomal protein S16B
rps19-2		40S ribosomal protein S19B/S19.2
rps2202		40S ribosomal protein S15a or S22
rps4-3		Protein with high similarity to ribosomal protein S4 (*S. cerevisiae* Rps4ap), member of the ribosomal S4e family, which are part of the small ribosomal subunit, and of the KOW (Kyprides-Ouzounis-Woese) motif family, contains an S4 domain
rrn3		Protein involved in initiation of transcription of rDNA promoter
sal3		Importin-beta homolog involved in Cdc25p nuclear import
scw1		Protein involved in negative regulation of cell wall integrity and septum formation, probably indirectly through a role in mRNA regulation
sds23		CBS-domain protein likely to be involved in cell cycle progression
sec21		Member of the adaptin N-terminal region-containing family, has moderate similarity to coatomer protein complex subunit gamma 1 (mouse Copg1), which is a subunit of the COP I vesicular coatomer complex and may mediate binding to membrane proteins
smg1		Protein with high similarity to sm core protein G (human SNRPG), which is a component of spliceosomal snRNPs that is involved in snRNP formation and autoantibodies are associated with systemic lupus erythematosus, contains an LSM domain
SPAC12G12.06c		Probable RNA 3'-terminal phosphate cyclase
SPAC1399.01c		Member of the xanthine or uracil permeases family of membrane transporters, has weak similarity to sodium-dependent vitamin C transporter 1 (solute carrier family 23 member 2, human SLC23A2), which acts in high-affinity ascorbate transport
SPAC13F5.03c		Member of the iron-containing alcohol dehydrogenase family, which oxidize ethanol to acetaldehyde with concomitant reduction of NAD
SPAC13G7.12c		Member of the choline or ethanolamine kinase family, which catalyze the formation of phosphatidylcholine by the CDP-choline pathway, and of the choline kinase N terminus containing family, has low similarity to choline kinase (*S. cerevisiae* Cki1p)
SPAC16C9.03		Protein with high similarity to *S. cerevisiae* Nmd3p, which is involved in the nuclear export of 60S ribosomal subunits, member of the NMD3 family
SPAC1786.02		Member of the lysophospholipase catalytic domain containing family, has moderate similarity to phospholipase B (*S. pombe* Plb1p), which preferentially deacylates phosphatidylinositol
SPAC1805.02c		Protein with high similarity to electron transfer flavoprotein beta subunit (human ETFB), which shuttles electrons and is associated with type II glutaric acidemia, member of the electron transfer flavoprotein (ETF) beta subunit family

SPAC18B11.04	Protein with high similarity to frequenin homolog (Drosophila) (human FREQ), which is a calcium binding protein and a putative kinase inhibitor that may have a regulatory role in secretion, contains three EF hand domains
SPAC18G6.09c	Protein of unknown function
SPAC19D5.04	Protein containing a HECT domain, which may confer ubiquitin-protein ligase activity, has low similarity to *S. cerevisiae* Tom1p, which is a protein required for the G2/M transition
SPAC1B1.04c	Protein with weak similarity to *S. cerevisiae* Pan3p, which is a component of *S. cerevisiae* Pab1p-stimulated poly(A) ribonuclease
SPAC1D4.03c	Member of the SAND family, has low similarity to uncharacterized human MGC13272
SPAC1F12.03c	Protein of unknown function
SPAC1F3.08c	Protein of unknown function
SPAC1F8.07c	Protein containing a thiamine pyrophosphate enzyme N-terminal TPP binding domain, C-terminal TPP binding domain, and central domain, which includes a 2-fold Rossman fold, has low similarity to pyruvate decarboxylase isozyme 2 *(S. cerevisiae* Pdc5p)
SPAC23A1.01c	Protein of unknown function, has low similarity to a region of podocalyxin like (human PODXL), which is a transmembrane sialomucin that is an L selectin ligand important for lymphocyte
SPAC23D3.12	Member of the sugar (and other) transporter family, has moderate similarity to *S. cerevisiae* Pho84p, which is a high-affinity inorganic phosphate/H+ symporter
SPAC23G3.07c	Protein of unknown function
SPAC26F1.0 CESR	*Protein with high similarity to aldehyde reductase (human AKR1A1), which is an NADPH-dependent aldo-keto reductase that reduces carbonyl-containing substrates and metabolizes xenobiotics, contains an aldo-keto reductase family domain*
SPAC27D7.1 CESR	*Protein of unknown function, has high similarity to uncharacterized S. pombe Spac27d7.10cp*
SPAC27F1.06c	Protein containing a FKBP-type peptidyl-prolyl cis-trans isomerase domain, has moderate similarity to *S. cerevisiae* Fpr3p, which is a peptidylprolyl cis-trans isomerase (PPIase) of the nucleolus that has FK506- and rapamycin-binding activity
SPAC29A4.16	Protein containing one protein kinase domain, has moderate similarity to *S. cerevisiae* Sat4p, which is a serine/threonine protein kinase involved in sensitivity to salt
SPAC29B12.05c	Protein of unknown function, has moderate similarity to uncharacterized *C. albicans* Ipf20015p
SPAC29B12.08	Protein of unknown function
SPAC2C4.18;SPAC25G	Protein containing an RNA recognition motif (RRM, RBD, or RNP), has a region of low similarity to a region of splicing factor arginine serine rich 10 (human SFRS10), which binds splicing enhancer elements and activates pre-mRNA splicing
SPAC30.10c	Protein with high similarity to cysteinyl-tRNA synthetase (*S. cerevisiae* Ynl247p), member of the class I cysteinyl (C) tRNA synthetase family
SPAC3C7.13 CESR	*Protein containing a glucose-6-phosphate dehydrogenase C-terminal domain and glucose-6-phosphate dehydrogenase NAD binding domain, has moderate similarity to glucose-6-phosphate dehydrogenase (S. cerevisiae Zwf1p)*
SPAC3F10.11c	Putative ABC multidrug or ion efflux transporter
SPAC3G6.05	Member of the Mpv17 and PMP22 family, which are peroxisomal proteins, has low similarity to mpv17 transgene (kidney disease mutant, mouse Mpv17), which is a transcriptional repressor that is associated with glomerulosclerosis and inner ear defects
SPAC4A8.10	Protein of unknown function, has low similarity to uncharacterized *S. cerevisiae* Rog1p
SPAC513.07 CESR	*Protein with moderate similarity to alpha-acetoxy ketone reductase (S. cerevisiae Gre2p), which is involved in diamide tolerance and induced by osmotic stress*
SPAC56E4.01c	Member of the protein phosphatase 2C family, which are Mg2+ dependent protein serine and threonine phosphatases, has moderate similarity to a region of *S. cerevisiae* Ptc5p
SPAC56E4.03	Protein with moderate similarity to aromatic amino acid aminotransferase I (*S. cerevisiae* Aro8p)
SPAC5H10.06c	Protein with high similarity to alcohol dehydrogenase IV (*S. cerevisiae* Adh4p), which is involved in carbohydrate metabolism, member of the iron-containing alcohol dehydrogenase family, which oxidize ethanol to acetaldehyde
SPAC644.08	Protein containing a haloacid dehalogenase-like hydrolase domain, has moderate similarity to uncharacterized *S. cerevisiae* Utr4p
SPAC8C9.07	Protein of unknown function
SPAC8C9.12c	Protein with high similarity to mitochondrial RNA splicing protein 3 (*S. cerevisiae* Mrs3p), which is associated with suppression of mitochondrial splicing defects, member of the mitochondrial carrier protein family of membrane transporters
SPACUNK4.10	Putative 2-hydroxyacid dehydrogenase
SPBC115.03	Protein containing a GFO, IDH, or MOCA oxidoreductase C-terminal alpha or beta domain and a NAD-binding Rossmann fold, which are found in some oxidoreductases that utilize NADP or NAD, has high similarity to uncharacterized S. pombe Spac26h5.09cp
SPBC119.10	Asparagine synthetase

SPBC11G11.03	Protein with high similarity to *S. cerevisiae* Mrt4p, which is involved in mRNA turnover, member of the ribosomal L10 family, which are part of the large ribosomal subunit
SPBC1604.04	Member of the mitochondrial carrier protein family of membrane transporters, has low similarity to solute carrier family 25 member 19 (human SLC25A19), which is a nucleotide transporter
SPBC1683.01	Putative inorganic phosphate transporter, belongs to MFS family of transporters
SPBC16A3.0 CESR	*Member of the zinc-binding dehydrogenase family, which catalyze reversible oxidation of ethanol to acetaldehyde, has low similarity to NOGO-interacting mitochondrial protein (human RTN4IP1), which binds human RTN4, human UQCRC2 and human UQCRC1*
SPBC16D10.06	Member of the ZIP zinc transporter family, which may be metal transporters, has moderate similarity to *S. cerevisiae* Zrt1p, which is a high-affinity zinc transport protein
SPBC1773.02c	Protein containing an AhpC or TSA family domain, has moderate similarity to *S. cerevisiae* Dot5p, which is a nuclear thiol peroxidase involved in telomeric silencing
SPBC17D11.03c	Member of the bacterial carboxymuconolactone decarboxylase family, which are involved in protocatechuate catabolism
SPBC19F5.02c	Protein containing six WD domains (WD-40 repeat), which may mediate protein-protein interactions, has moderate similarity to *S. cerevisiae* Utp4p, which is a component of the U3 snoRNP (also called small subunit processome) required for 18S rRNA biogenesis
SPBC19F5.03	Member of the SacI homology domain containing family, has moderate similarity to *S. cerevisiae* Sac1p, which is a polyphosphoinositide phosphatase that plays a role in Golgi function and actin cytoskeleton organization
SPBC1A4.07c	Protein with high similarity to *S. cerevisiae* Sof1p, which is a protein associated with U3 small nucleolar RNA (snoRNA) that is involved in 18S pre-rRNA maturation, member of the Sof1-like domain containing family and contains seven WD domains
SPBC215.10	Protein containing a haloacid dehalogenase-like hydrolase domain, has high similarity to uncharacterized *S. pombe* Spac25b8.12cp
SPBC216.03	Protein of unknown function, has low similarity to uncharacterized *S. cerevisiae* Ymr090p, which is a putative oxidoreductase that may play a role in energy pathways
SPBC21C3.09c	Protein with similarity to prokaryotic 2-hydroxyhepta-2,4-diene-1,7-dioate isomerase
SPBC21C3.12c	Protein of unknown function
SPBC21D10.09c	Protein containing a C3HC4 type (RING) zinc finger, which may mediate protein-protein interactions, has weak similarity to uncharacterized *S. cerevisiae* Ymr247p
SPBC23E6.06c	Member of the 3,4-dihydroxy-2-butanone 4-phosphate (DHBP) synthase family, which are involved in riboflavin biosynthesis and may be part of a bifunctional enzyme, has high similarity to uncharacterized C. albicans Rib3p
SPBC25B2.07c	Serine- and proline-rich protein of unknown function, appears to localize to microtubules
SPBC26H8.08c	Member of the uncharacterized GTPase family, has moderate similarity to *S. cerevisiae* Nug1p, which is a nuclear protein required for 60S ribosomal subunit export from the nucleus
SPBC29A3.01	Member of the E1-E2 (P-type) ATPase and the haloacid dehalogenase or epoxide hydrolase families, contains a heavy-metal-associated domain, has moderate similarity to *S. cerevisiae* Ccc2p, which is a copper-transporting P-type ATPase
SPBC336.13c	Member of the signal peptidases type I family, which cleave signal peptides from secreted proteins, has moderate similarity to *S. cerevisiae* Imp2p, which is a catalytic subunit of the mitochondrial inner membrane protease S. cerevisiae Impp
SPBC337.10c	Member of the DUF689 domain of unknown function family, has low similarity to uncharacterized mouse 2810413N20Rik
SPBC3H7.08c	Protein of unknown function
SPBC4B4.10c	Member of the autophagy protein Apg5 family, has low similarity to autophagy 5 like (apoptosis specific protein, mouse Apg5l), which may have a role in membrane dynamics
SPBC4F6.09	Protein with low similarity to *C. albicans* Arn1p, which is involved in the transport of ferrichrome-iron and in invasion of epithelial cells
SPBC56F2.08c	Protein containing five Pumilio-family (Puf) RNA binding domains and an RNA recognition motif (RRM, RBD, or RNP), has low similarity to a region of *S. cerevisiae* Jsn1p, which suppresses the hyperstable microtubule phenotype of tub2-150 when overproduced
SPBC609.04	Protein with moderate similarity to *C. dubliniensis* Mdr1p, which is a multidrug transporter that is involved in drug resistance
SPBC646.10c	Protein with high similarity to *S. cerevisiae* Sik1p, which is a nucleolar protein component of box C-D snoRNPs and is necessary for 2'-O-methylation of ribosomal RNAs, member of the putative snoRNA binding domain containing family
SPBC651.09c	Protein containing a plus-3 domain, which may be involved in DNA binding, has low similarity to *S. cerevisiae* Rtf1p, which is important for TATA site selection by S. cerevisiae Spt15p and is involved in regulation of transcription elongation
SPBC660.06 CESR	*Protein of unknown function, has low similarity to uncharacterized S. pombe Pi003p*

SPBC660.15	Protein containing an RNA recognition motif (RRM, RBD, or RNP), has low similarity to nuclear polyadenylated RNA-binding protein (*S. cerevisiae* Hrp1p), which is a nuclease that binds 3' UTR
SPBC725.01	Protein with high similarity to mitochondrial aspartate aminotransferase (glutamic oxaloacetic transaminase, mouse Got2), which transfers the aspartate amino group to 2-oxoglutarate to form oxaloacetate, contains an aminotransferase class I and II domain
SPBC887.17	Member of the xanthine or uracil permeases family of membrane transporters
SPBC8E4.02c	Protein of unknown function
SPBP4G3.01	Member of the sugar (and other) transporter family, has moderate similarity to *S. cerevisiae* Pho84p, which is a high-affinity inorganic phosphate/H+ symporter
SPBP8B7.05c	Protein containing a carbonic anhydrase domain, has low similarity to *S. cerevisiae* Nce103p, which is involved in protection against oxidative damage
SPBPB8B6	Member of the amidase family, which catalyze hydrolysis of amides, has low similarity to fatty acid amide hydrolase (rat Faah), which is an integral membrane serine hydrolase that degrades neuromodulatory fatty acid amides
SPCC1322.14c	Protein that may be involved in phosphate metabolism
SPCC1450.01c	Protein of unknown function
SPCC1682.08c	Protein containing six Pumilio-family (Puf) RNA binding domains, has a region of low similarity to a region of *S. cerevisiae* Puf3p, which is involved in mRNA metabolism
SPCC1742.01	Protein with weak similarity to agglutinin-like adhesin (*C. albicans* Als5p), which is involved in adhesion to extracellular matrix proteins
SPCC24B10.14c	Protein of unknown function
SPCC2H8.02	Member of the sugar (and other) transporter family, has low similarity to *S. cerevisiae* Pho84p, which is a high-affinity inorganic phosphate:H+ symporter
SPCC364.07	Protein with high similarity to 3-phosphoglycerate dehydrogenase (*S. cerevisiae* Ser33p), which catalyzes the first step in the synthesis of serine, contains D-isomer specific 2-hydroxyacid dehydrogenase catalytic and NAD binding domains and an ACT domain
SPCC553.09c	Member of the DNA polymerase alpha subunit B family, has low similarity to DNA polymerase alpha 86 kDa subunit (*S. cerevisiae* Pol12p), which is the B subunit of polymerase alpha-primase
SPCC63.14 CESR	*Protein of unknown function, appears to localize to the cytoplasm*
SPCC736.03c	Protein containing a tRNA synthetases class II core domain (F) domain and a ferredoxin-fold anticodon binding domain, has moderate similarity to mitochondrial phenylalanyl-tRNA synthetase (*S. cerevisiae* Msf1p)
SPCC757.11c	Protein of unknown function, has moderate similarity to uncharacterized *S. pombe*
SPCP31B10.09	Protein containing two C2 domains, which may be involved in calcium-dependent phospholipid binding, has low similarity to uncharacterized *S. pombe* Spapyuk71.03cp
spf38	Component of the Cdc5p-associated complex, possibly involved in pre-mRNA splicing
spp42	Probable spliceosomal subunit, component of a 40S snRNP-containing complex; similar to *S. cerevisiae* Prp8p
ste13	Putative ATP-dependent RNA helicase
stm1	G protein-coupled receptor that acts as a nutrient sensor for sexual diffentiation and stress response pathways
sup35	Omnipotent nonsense suppressor, EF1 alpha factor-like GTP-binding protein
tel1	Phosphotidylinositol kinase involved in telomere length control in parallel with the Radp-dependent pathway
tf2-10	Pseudogene
tf2-3: tf2-4	Transposable element
tf2-8	Transposable element
thi2	Thiazole biosynthetic enzyme
tif35	Putative translation initiation factor 3, RNA-binding subunit
tif6	Protein with high similarity to translation initiation factor 6 (eIF6, *S. cerevisiae* Cdc95p), which is a required for transit of pre-60S ribosomal subunits to the cytoplasm, member of the eukaryotic translation initation factor 6 (eIF-6) family
ubc15	Putative ubiquitin-conjugating enzyme that is involved in negative regulation of heterochromatin silencing by affecting histone H3 methylation
ucp9	Protein with high similarity to *S. cerevisiae* Snf1p, which is a serine/threonine protein kinase essential for derepression of glucose-repressed genes, contains one protein kinase domain
ura3	Dihydroorotate dehydrogenase precursor
aar2	Protein of unknown function, has weak similarity to uncharacterized human C20orf4
