# Supplementary material for: Global transcriptional response after exposure of fission yeast cells to ultraviolet light
Source: BMC Cell Biol. 2009 Dec 16;10:87. doi: 10.1186/1471-2121-10-87 (PMC2806298; doi:10.1186/1471-2121-10-87)
Supplement: Additional file 5 — Enriched gene ontology groups in the restrictive-temperature experiment. Gene ontology (GO) enrichment analysis was performed on the 43 UVC-induced genes in the restrictive-temperature experiment (Table 1) using the DAVID software after ID-conversion. Ten unique genes (20%) were members of enriched GO groups. [file 1471-2121-10-87-S5.PDF]

**Enriched Gene Ontology Groups in the restrictive-temperature experiment**

| Category | GO number | Term | Count* | % | P-value |
|---|---|---|---|---|---|
| GOTERM_Cellular Component | GO:0005887 | integral to plasma membrane | 3 | 8.11% | 0,024 |
| | GO:0044459 | plasma membrane part | 4 | 10.81% | 0,035 |
| | GO:0031226 | intrinsic to plasma membrane | 3 | 8.11% | 0,038 |
| GOTERM_Biological Process | GO:0015674 | di-, tri-valent inorganic cation transport | 4 | 10.81% | 0,001 |
| | GO:0006812 | cation transport | 5 | 13.51% | 0,007 |
| | GO:0030001 | metal ion transport | 4 | 10.81% | 0,008 |
| | GO:0030003 | cellular cation homeostasis | 4 | 10.81% | 0,018 |
| | GO:0055082 | cellular chemical homeostasis | 4 | 10.81% | 0,020 |
| | GO:0050801 | ion homeostasis | 4 | 10.81% | 0,022 |
| GOTERM_Molecular Function | GO:0008324 | cation transmembrane transporter activity | 5 | 13.51% | 0,011 |
| | GO:0046873 | metal ion transmembrane transporter activity | 3 | 8.11% | 0,042 |

* Count denotes the number of genes in our dataset in each cluster.

Percent coverage of these genes relative to the numbers on the gene ontology clusters was calculated.
