# Supplementary material for: Global transcriptional response after exposure of fission yeast cells to ultraviolet light
Source: BMC Cell Biol. 2009 Dec 16;10:87. doi: 10.1186/1471-2121-10-87 (PMC2806298; doi:10.1186/1471-2121-10-87)
Supplement: Additional file 7 — Genes induced in the time-course experiment: protein-protein interactions. Gene products of the regulated genes from the timecourse experiment form an interconnected network involving translation and transcription. Protein-protein interactions were analyzed in FunCoup using the corresponding S. cerevisiae orthologues. [file 1471-2121-10-87-S7.PDF]

Protein	Description	Interacting partner(s)	Degree of protein-protein interaction	Citation
AAR2 (aar2)	A1 cistron-splicing factor	BRR2 (brr2)	Strong	[2]
		PRP8 (spp42)	Strong	[1, 2]
BRR2 (brr2)	Pre-mRNA-splicing helicase	PRP8 (spp42)	Strong	[2-4]
		AAR2 (aar2)	Strong	[2]
		K6PF2 (pfk1)	Strong	[2]
		RUXG (smg1)	Moderate	[2, 4]
		RL5 (rpl5-2)	Strong	[2]
ENO2 (eno1)	Enolase 2 (2-phosphoglycerate dehydratase 2)	K6PF2 (pfk1)	Strong	[2,5]
		RL5 (rpl5-2)	Strong	[2, 6]
		RL24B (rpl24-2)	Moderate	IntAct, HPRD, BIND databases
K6PF2 (pfk1)	6-phosphofructokinase subunit beta	ENO2 (eno1)	Strong	[2, 5]
		RL5 (rpl5-2)	Strong	[2]
		BRR2 (brr2)	Strong	[2]
		PYRD (ura3)	Strong	[7]
MRT4 (SPBC11G11.03)	mRNA turnover protein 4.	RL5 (rpl5-2)	Strong	[2]
		RS16 (rps16-2)	Moderate	[2]
PRP8 (spp42)	Pre-mRNA-splicing factor 8.	BRR2 (brr2)	Strong	[2-4]
		RUXG (smg1)	Moderate	[2, 4]
		AAR2 (aar2)	Strong	[2]
		RS16 (rps16-2)	Moderate	[2]
		RL5 (rpl5-2)	Strong	[2]
PYRD (ura3)	Dihydroorotate dehydrogenase	K6PF2 (pfk1)	Strong	[7]
RL24B (rpl24-2)	60S ribosomal protein L24-B	RS16 (rps16-2)	Moderate	IntAct, HPRD, BIND databases
		RS2 (rps001)	Moderate	[5]
		ENO2 (eno1)	Moderate	IntAct, HPRD, BIND databases
RL5 (rpl5-2)	60S ribosomal protein L5	ENO2 (eno1)	Strong	[2, 6]
		RS16 (rps16-2)	Strong	[2]
		RS2 (rps001)	Strong	[5, 6, 8]
		K6PF2 (pfk1)	Strong	[2]
		MRT4 (SPBC11G11.03)	Strong	[2]
		RS4 (rps4-3)	Strong	[2, 8]
		SIK1 (SPBC646.10c)	Strong	[2, 5, 9]
		RS14A (rps14-2)	Strong	[2]
		BRR2 (brr2)	Strong	[2]
		RPC5 (rpc40)	Strong	[2]
		PRP8 (spp42)	Strong	[2]
RPB10 (rpb10)	DNA-directed RNA polymerases I/II/III subunit 10	RPC5 (rpc40)	Strong	[1, 2]
		UTP4 (SPBC19F5.02c)	Moderate	[2]
		SIK1 (SPBC646.10c)	Moderate	[2]
		SOF1 (SPBC1A4.07c)	Strong	[2]
		RS16 (rps16-2)	Strong	[2]

		RS4 (rps4-3)	Strong	[2]
RPC10 (rpc10)	DNA-directed RNA polymerases I, II, and III 7.7 kDa polypeptide	RPC5 (rpc40)	Strong	IntAct, HPRD, BIND databases
RPC5 (rpc40)	DNA-directed RNA polymerases I and III 40 kDa polypeptide	RPB10 (rpb10)	Strong	[1, 2]
		RPC10 (rpc10)	Strong	IntAct, HPRD, BIND databases
		RS4 (rps4-3)	Strong	[2]
		RL5 (rpl5-2)	Strong	[2]
RS14A (rps14-2)	40S ribosomal protein S14-A	RS4 (rps4-3)	Strong	[2, 5, 9]
		RS16 (rps16-2)	Strong	[2]
		SOF1 (SPBC1A4.07c)	Strong	[2, 9]
		RS2 (rps001)	Strong	[5]
		SIK1 (SPBC646.10c)	Strong	[2, 5]
		RL5 (rpl5-2)	Strong	[2]
RS16 (rps16-2)	40S ribosomal protein S16	RL24B (rpl24-2)	Moderate	IntAct, HPRD, BIND databases
		SIK1 (SPBC646.10c)	Strong	[2]
		RL5 (rpl5-2)	Strong	[2]
		RS14A (rps14-2)	Strong	[2]
		RS4 (rps4-3)	Strong	[2]
		MRT4 (SPBC11G11.03)	Moderate	[2]
		SOF1 (SPBC1A4.07c)	Strong	[2]
		RPB10 (rpb10)	Strong	[2]
		PRP8 (spp42)	Moderate	[2]
RS2 (rps001)	40S ribosomal protein S2 (Omnipotent suppressor protein SUP44)	SIK1 (SPBC646.10c)	Moderate	[5]
		RL24B (rpl24-2)	Moderate	[5]
		RL5 (rpl5-2)	Strong	[2, 6, 8]
		RS4 (rps4-3)	Strong	[2, 5, 8]
		RS14A (rps14-2)	Strong	[5]
RS4 (rps4-3)	40S ribosomal protein S4	RS14A (rps14-2)	Strong	[2, 5, 9]
		RS16 (rps16-2)	Strong	[2]
		RS2 (rps001)	Strong	[2, 5, 8]
		SIK1 (SPBC646.10c)	Strong	[2, 5, 9]
		SOF1 (SPBC1A4.07c)	Strong	[2, 9]
		UTP4 (SPBC19F5.02c)	Strong	[2, 9]
		RPC5 (rpc40)	Strong	[2]
		RL5 (rpl5-2)	Strong	[2, 8]
		RPB10 (rpb10)	Strong	[2]
RUXG (smg1)	Small nuclear ribonucleoprotein G (snRNP-G)	PRP8 (spp42)	Moderate	[2, 4]
		BRR2 (brr2)	Moderate	[2, 4]
SIK1 (SPBC646.10c)	Protein SIK1 (Nucleolar protein NOP56)	SOF1 (SPBC1A4.07c)	Strong	[1, 2, 9]
		UTP4 (SPBC19F5.02c)	Strong	[1, 2, 9]
		RS16 (rps16-2)	Strong	[2]

		RS2 (rps001)	Moderate	[5]
		RS4 (rps4-3)	Strong	[2, 5, 9]
		RPB10 (rpb10)	Moderate	[2]
		RS14A (rps14-2)	Strong	[2, 5]
		RL5 (rpl5-2)	Strong	[2, 5, 9]
SOF1 (SPBC1A4.07c)	Protein SOF1	SIK1 (SPBC646.10c)	Strong	[1, 2, 9]
		UTP4 (SPBC19F5.02c)	Strong	[1, 2, 9, 10]
		RS14A (rps14-2)	Strong	[2, 9]
		RS4 (rps4-3)	Strong	[2, 9, 10]
		RPB10 (rpb10)	Strong	[2]
		RS16 (rps16-2)	Strong	[2]
UTP4 (SPBC19F5.02c)	U3 small nucleolar RNA-associated protein 4	SOF1 (SPBC1A4.07c)	Strong	[1, 2, 9, 10]
		SIK1 (SPBC646.10c)		[1, 2, 9]
		RPB10 (rpb10)	Moderate	[2]
		RS4 (rps4-3)	Strong	[2, 9, 10]

References for supplementary material

1. Gavin AC, Bosche M, Krause R, Grandi P, Marzioch M, Bauer A, Schultz J, Rick JM, Michon AM, Cruciat CM *et al*: **Functional organization of the yeast proteome by systematic analysis of protein complexes**. *Nature* 2002, **415**(6868):141-147.
2. Gavin AC, Aloy P, Grandi P, Krause R, Boesche M, Marzioch M, Rau C, Jensen LJ, Bastuck S, Dumpelfeld B *et al*: **Proteome survey reveals modularity of the yeast cell machinery**. *Nature* 2006, **440**(7084):631-636.
3. Hazbun TR, Malmstrom L, Anderson S, Graczyk BJ, Fox B, Riffle M, Sundin BA, Aranda JD, McDonald WH, Chiu CH *et al*: **Assigning function to yeast proteins by integration of technologies**. *Molecular cell* 2003, **12**(6):1353-1365.
4. Ohi MD, Link AJ, Ren L, Jennings JL, McDonald WH, Gould KL: **Proteomics analysis reveals stable multiprotein complexes in both fission and budding yeasts containing Myb-related Cdc5p/Cef1p, novel pre-mRNA splicing factors, and snRNAs**. *Mol Cell Biol* 2002, **22**(7):2011-2024.
5. Graumann J, Dunipace LA, Seol JH, McDonald WH, Yates JR, 3rd, Wold BJ, Deshaies RJ: **Applicability of tandem affinity purification MudPIT to pathway proteomics in yeast**. *Mol Cell Proteomics* 2004, **3**(3):226-237.
6. Guerrero C, Tagwerker C, Kaiser P, Huang L: **An integrated mass spectrometry-based proteomic approach: quantitative analysis of tandem affinity-purified in vivo cross-linked protein complexes (QTAX) to decipher the 26 S proteasome-interacting network**. *Mol Cell Proteomics* 2006, **5**(2):366-378.
7. Ho Y, Gruhler A, Heilbut A, Bader GD, Moore L, Adams SL, Millar A, Taylor P, Bennett K, Boutilier K *et al*: **Systematic identification of protein complexes in Saccharomyces cerevisiae by mass spectrometry**. *Nature* 2002, **415**(6868):180-183.
8. Harnpicharnchai P, Jakovljevic J, Horsey E, Miles T, Roman J, Rout M, Meagher D, Imai B, Guo Y, Brame CJ *et al*: **Composition and functional characterization of yeast 66S ribosome assembly intermediates**. *Molecular cell* 2001, **8**(3):505-515.
9. Grandi P, Rybin V, Bassler J, Petfalski E, Strauss D, Marzioch M, Schafer T, Kuster B, Tschochner H, Tollervey D *et al*: **90S pre-ribosomes include the 35S pre-rRNA, the U3 snoRNP, and 40S subunit processing factors but predominantly lack 60S synthesis factors**. *Molecular cell* 2002, **10**(1):105-115.
10. Rempola B, Karkusiewicz I, Piekarska I, Rytka J: **Fcf1p and Fcf2p are novel nucleolar Saccharomyces cerevisiae proteins involved in pre-rRNA processing**. *Biochem Biophys Res Commun* 2006, **346**(2):546-554.
