# Supplementary material for: Global transcriptional response after exposure of fission yeast cells to ultraviolet light
Source: BMC Cell Biol. 2009 Dec 16;10:87. doi: 10.1186/1471-2121-10-87 (PMC2806298; doi:10.1186/1471-2121-10-87)
Supplement: Additional file 6 — Fission yeast homologues of UPR-induced budding yeast genes. We used the data from both the restrictive-temperature and timecourse experiments to investigate whether UPR genes are induced by UVC in fission yeast. We identified UPR-genes in fission yeast as the homologues of the UPR-induced budding yeast genes. [file 1471-2121-10-87-S6.PDF]

Fission yeast homologues of UPR-induced budding yeast genes

Gene annotations are from GeneDB http://www.genedb.org/genedb/pombe/index

The putative homologues were identified using http://www.ihop-net.org/UniPub/i h

	Time-course experiment													
	**Time (min)**	**0**	**30**	**90**	**0**	**30**	**90**	**0**	**30**	**90**	**0**	**30**	**90**	
	**Experiment**	**UV**	**UV**	**UV**	**UV**	**UV**	**UV**	**control**	**control**	**control**	**control**	**control**	**control**	
	**Biological repeat**	**1**	**1**	**1**	**2**	**2**	**2**	**1**	**1**	**1**	**2**	**2**	**2**	
**Budding yeast**	**Fission yeast**	**Normalised expression values compared to C0**												**Annotation**
**IRE1**	***SPAC167.01***	1,037	1,446	1,135	1,119		1,212	1	1,945	0,962	1	1,280	0,878	Protein containing a protein kinase domain, has low similarity to S. cerevisiae Ire1p, which is a protein kinase and endoribonuclease involved in the unfolded protein response
**PDI1**	***SPAC1F5.02***	0,847	1,173	1,365	0,854	1,014	1,048	1	0,925	1,176	1	0,996	1,200	Protein containing two thioredoxin domains, has moderate similarity to A. niger PdiA, which is a putative protein disulfide isomerase that may have a role in secretion
**LHS1**	***SPAC1F5.06***	0,830		0,977	0,972	1,053	1,025	1	1,168	0,862	1	1,033	1,146	Member of the Hsp70 family, which are stress-induced protein chaperones, has low similarity to oxygen regulated protein 150 kDa (human HYOU1), which is a putative endoplasmic reticulum chaperone for angiogenic factors that is induced by hypoxia
**KAR2**	***bip***	0,930		0,880	1,328	0,679	0,727	1	0,549	0,627	1	0,632	0,706	Binding protein (BiP) homolog involved in protein folding in the endoplasmic reticulum
**SEC61**	***sec61***	1,154	0,928	1,091				1	1,135	0,939				Essential protein, has high similarity to S. cerevisiae Sec61p
**KTR1**	***SPBC19C7.12c***		1,395	1,258				1	1,247	1,054				Putative 2-alpha-mannosyltransferase
**ALG7**	***gpt***	0,784		0,592	0,897	0,964	0,816	1	0,685	0,712	1		0,966	Dolichol phosphate-dependent N-acetylglucosamine-1-P transferase (GPT)
**STE24**	***SPAC3H1.05***		0,718	0,823	1,038	1,227	1,083	1	0,719	0,651	1	1,097	0,904	Member of the M48 peptidase family of zinc metalloproteases, has moderate similarity to S. cerevisiae Ste24p, which is a prenyl-dependent protease involved in N-terminal proteolytic and C-terminal CAAX processing of a-factor (S. cerevisiae Mfa1p)
**RAM2**	***cwp1***	1,113		1,075	0,928	0,951	0,910	1	1,841	0,733	1	1,379		Alpha subunit of both geranylgeranyltransferase I (GGTase I) and farnesyltransferase (FTase), involved in protein prenylation
**PMT1**	***SPAC22A12.07c***	0,961	0,920	0,904	0,773	0,678	0,746	1	0,882	0,713	1	0,706	0,618	Member of the dolichyl-phosphate-mannose-protein mannosyltransferase family, contains three MIR (protein mannosyltransferase, IP3R and RyR) domains, has low similarity to mannosyltransferase (C. albicans Pmt1p), which acts in O-glycosylation
**PMT2**	***SPAPB1E7.09***	0,748	0,780	0,856	0,864	0,832	0,668	1	0,774	0,903	1	1,178		Member of the dolichyl-phosphate-mannose-protein mannosyltransferase family, contains three MIR (protein mannosyltransferase, IP3R and RyR) domains, has moderate similarity to mannosyltransferase (S. cerevisiae Pmt3p), which acts in O-glycosylation
**RIB1**	***SPAP27G11.09c***	0,985	1,099	1,863	1,032	1,366	1,375	1	1,030	1,069	1	0,872	0,958	Protein with high similarity to GTP cyclohydrolase II (S. cerevisiae Rib1p), which is the initial and rate-limiting step in the riboflavin biosynthesis pathway, member of the GTP cyclohydrolase II family, which are involved in riboflavin biosynthesis

	**Restrictive-temperature experiment**			
	**Repeat**	**1**	**2**	
**Budding yeast**	**Fission yeast**	**UV/C**	**UV/C**	**Annotation**
**IRE1**	***SPAC167.01***	1,225	1,252	Member of the ribonuclease 2-5A family, contains a protein kinase domain, has low similarity to S. cerevisiae Ire1p, which is a protein kinase and a site-specific endoribonuclease that is involved in the unfolded protein response
**PDI1**	***SPAC1F5.02***	0,784	0,852	Protein containing two thioredoxin domains, has moderate similarity to A. niger PdiA, which is a putative protein disulfide isomerase that may have a role in secretion
**LHS1**	***SPAC1F5.06***	0,685	0,757	Member of the Hsp70 family, which are stress-induced protein chaperones, has low similarity to oxygen regulated protein 150 kDa (human HYOU1), which is a putative endoplasmic reticulum chaperone for angiogenic factors that is induced by hypoxia
**ERO1**	***SPCC1450.14c***	1,492	1,385	Member of the endoplasmic reticulum oxidoreductin 1 (ERO1) family, which are involved in the formation of disulfide bonds, has low similarity to S. cerevisiae Ero1p, which is required for protein disulfide bond formation in the endoplasmic reticulum
**KAR2**	***bip***	0,773	0,863	Binding protein (BiP) homolog involved in protein folding in the endoplasmic reticulum
**SEC61**	***sec61***	0,868	0,889	Essential protein, has high similarity to S. cerevisiae Sec61p
**ALG6**	***SPBC342.01c***	0,853	0,991	Putative alpha-1,3-glucosyltransferase involved in synthesis of the dolichol-linked, oligosaccharide
**GAA1**	***SPAC1002.11***	1,249	1,057	Member of the Gaa1-like, GPI transamidase component family, has low similarity to S. cerevisiae Gaa1p, which is a subunit of a glycosylphosphatidylinositol (GPI) transamidase and involved in the attachment of GPI anchors to proteins
**KTR1**	***SPBC19C7.12c***	1,154	1,167	Putative 2-alpha-mannosyltransferase
**ALG7**	***gpt***	0,896	0,908	Dolichol phosphate-dependent N-acetylglucosamine-1-P transferase (GPT)
**STE24**	***SPAC3H1.05***	1,076	1,126	Member of the M48 peptidase family of zinc metalloproteases, has moderate similarity to S. cerevisiae Ste24p, which is a prenyl-dependent protease involved in N-terminal proteolytic and C-terminal CAAX processing of a-factor (S. cerevisiae Mfa1p)
**RAM2**	***cwp1***	0,642	0,677	Alpha subunit of both geranylgeranyltransferase I (GGTase I) and farnesyltransferase (FTase), involved in protein prenylation
**PMT1**	***SPAC22A12.07c***	0,76	0,766	Member of the dolichyl-phosphate-mannose-protein mannosyltransferase family, which catalyze O-linked glycosylation of proteins, and the protein mannosyltransferase, IP3R and RyR (MIR) domain containing family, has low similarity to C. albicans Pmt1p
**PMT2**	***SPAPB1E7.09***	0,778	0,831	Member of the dolichyl-phosphate-mannose-protein mannosyltransferase and the protein mannosyltransferase, IP3R and RyR (MIR) domain containing families, has moderate similarity to mannosyltransferase (S. cerevisiae Pmt3p), which acts in O-glycosylation
**RIB1**	***SPAP27G11.09c***	1,011	1,054	Protein with high similarity to GTP cyclohydrolase II (S. cerevisiae Rib1p), which is the initial and rate-limiting step in the riboflavin biosynthesis pathway, member of the GTP cyclohydrolase II family, which are involved in riboflavin biosynthesis

Fission yeast homologues of 47 UPR genes in budding yeast were identified and their expression levels after UV irradiation were compared to those in unirradiated control cells.
